# Transcriptomic Analysis of Hepatitis B Infected Liver for Prediction of Hepatocellular Carcinoma

**DOI:** 10.3390/biology12020188

**Published:** 2023-01-26

**Authors:** Diren Arda Karaoglu, Meral Uner, Cem Simsek, Ali Osmay Gure, Secil Demirkol-Canli

**Affiliations:** 1 Faculty of Medicine, Hacettepe University, 06100 Ankara, Turkey; 2 Department of Pathology, Hacettepe University Faculty of Medicine, Sıhhiye, 06100 Ankara, Turkey; 3 Department of Gastroenterology, Hacettepe University Faculty of Medicine, 06100 Ankara, Turkey; 4 Department of Medical Biology, Acibadem University, 34684 Istanbul, Turkey; 5 Molecular Pathology Application and Research Center, Hacettepe University, 06100 Ankara, Turkey; 6 Tumor Pathology, Cancer Institute, Hacettepe University, 06100 Ankara, Turkey

**Keywords:** HBV, hepatitis, hepatocellular cancer, biomarkers, transcriptomics

## Abstract

**Simple Summary:**

Hepatitis B virus infection increases the risk of developing liver cancer, a common health problem worldwide. In this study, we aimed to identify patients with hepatitis B infection who have a higher risk of cancer development. Our results reveal that liver tissues from hepatitis B patients can be clearly classified according to whether they express higher levels of messenger RNA produced from a panel of genes related to proliferation and inflammation. A subgroup of genes in this panel are linked to cancer development and shorter lifetimes in liver cancer patients. The new gene list we define here has the potential to be used as a new approach for the molecular identification of patients with hepatitis-B-virus-infected livers and who are at risk of liver cancer development.

**Abstract:**

Hepatocellular cancer (HCC) is a leading cause of cancer-related mortality worldwide, and chronic hepatitis B virus infection (CHB) has been a major risk factor for HCC development. The pathogenesis of HBV-related HCC has been a major focus revealing the interplay of a multitude of intracellular signaling pathways, yet the precise mechanisms and their implementations to clinical practice remain to be elucidated. This study utilizes publicly available transcriptomic data from the livers of CHB patients in order to identify a population with a higher risk of malignant transformation. We report the identification of a novel list of genes (PCM1) which can generate clear transcriptomic sub-groups among HBV-infected livers. PCM1 includes genes related to cell cycle activity and liver cancer development. In addition, markers of inflammation, M1 macrophages and gamma delta T cell infiltration are present within the signature. Genes within PCM1 are also able to differentiate HCC from normal liver, and some genes within the signature are associated with poor prognosis of HCC at the mRNA level. The analysis of the immunohistochemical stainings validated that proteins coded by a group of PCM1 genes were overexpressed in liver cancer, while minimal or no expression was detected in normal liver. Altogether, our findings suggest that PCM1 can be developed into a clinically applicable method to identify CHB patients with a higher risk of HCC development.

## 1. Introduction

Hepatocellular cancer (HCC) is the sixth most commonly diagnosed cancer and fourth leading cause of cancer-related mortality worldwide [1]. Chronic heavy alcohol consumption, non-alcoholic fatty liver disease (NAFLD), chronic infection with viruses, chronic liver diseases such as chronic biliary disease, genetic or metabolic liver diseases and exposure to toxic chemicals such as aflatoxins are critical etiological factors in HCC [2]. However, hepatitis B virus (HBV) is the main risk factor in approximately one half of HCC cases [3], and 20–25% of the patients with chronic hepatitis B (CHB) infection have a risk of death from HCC [4]. As mortality due to HBV is increasing [5], a better definition of risk factors and the elucidation of the molecular mechanisms related to HCC pathogenesis are needed.

One of the major players in HCC pathogenesis in CHB is inflammation that eventually progresses to cirrhosis [6]. Detecting patients with increased inflammation is critical, since immediate treatment is recommended in such cases to delay and possibly reverse progression to cirrhosis and eventually to HCC [7]. Although aminotransferase (ALT) and viral nucleic acid levels are used to predict prognosis in CHB patients, neither is considered sensitive enough to guide antiviral treatment per se, as there is a considerable volume of patients with hepatic necroinflammation and severe hepatic fibrosis with neither detectable HBV DNA nor elevated ALT levels [8]. Various other potential clinical indicators and scores of disease progression have been studied, such as HBV genotypes, naturally occurring HBV mutants and the presence of hepatic steatosis, which are not yet sufficient to completely explain clinical outcomes [9]. Multiple risk prediction models based on clinical factors such as age, sex, albumin, bilirubin, HBV DNA, ALT, HBeAg, alcohol history and liver stiffness for HBV-related HCC have also been proposed [10] but have limited predictive values (AUCs of 0.72–0.82 and 0.72–0.75) for the prediction of the development of HCC at 3 years and 5 years, respectively [11]. Therefore, there remain other biological factors involved in the progression of CHB which need to be explored in order to improve the management of CHB. The importance of the virus integration sites in the host genome is also debatable. Several cases were reported in which HBV integration was detected in non-tumor tissues but not in the paired tumor, indicating that HBV integration may not always be a driver of carcinogenesis; furthermore, there are also cases in which the viral integration sites are not known drivers of liver cancer [12].

Previous studies revealed large heterogeneity of gene expression profiles following HBV infection or HBx transfection, affecting multiple carcinogenic signaling pathways [13,14]. In this study, we used an unsupervised transcriptomic approach to define sub-groups of CHB patients with a high-risk of developing HCC, which could possibly be used to guide therapy as well as HCC screening. Our analysis identified a novel list consisting of 176 genes, which we name the “Proliferation, cancer and M1 macrophage (PCM1)” signature, that identifies two distinct sub-groups among livers with HBV infection. One of these groups, “PCM1-U (PCM1-upregulated)”, showed an increased expression of cell cycle-related genes and enrichment of liver cancer gene-sets, increased M1 macrophage fractions, T cell infiltration and proinflammatory responses. Such patients had generally high ALT and AST levels, and samples in this group were mostly at the immune clearance phase. The second group, “PCM1-D (PCM1-downregulated)”, was relatively heterogeneous in terms of ALT, AST, HBV DNA levels and the virus phase.

## 2. Materials and Methods

### 2.1. Study Cohorts and Microarray Data Processing

Gene expression data of all datasets were obtained from the GEO database [15] (accessed on 1 August 2019) (http://www.ncbi.nlm.nih.gov/geo/). Microarray data of 122, 124, 83 and 73 liver samples from CHB (other pathologies excluded) patients (GSE83148 [16], GSE84044 [17], GSE65359 [18] and GSE83898 [19], respectively) were used for the discovery and validation of the gene panel. Clinical data including ALT, AST levels and HBV DNA were obtained from series matrix files of GSE83148 and the supplementary data of Wang et al. [17]. Viral-phase information was extracted from the series matrix file of GSE65359. GSE14520 [20] (Affymetrix HT Human Genome U133A Platform-Testing group) and GSE121248 [21] datasets including gene expression data from HBV-related HCC and normal liver samples were utilized (GSE121248 = 70 HBV + HCC tumor, 37 adjacent normal tissue samples, GSE14520 = 212 paired HBV + HCC tumor-normal liver samples). Overall survival and status information of the 208 HCC patients in GSE14520 were obtained from the dataset’s series matrix file. All CEL files were normalized by BRB Array Tools using RMA method (https://brb.nci.nih.gov/BRB-ArrayTools/, version 4.6.1, accessed on 1 August 2019). GSE83898 [19] raw microarray data was quantile normalized and log2 transformed using the “lumi” R package [22]. Annotation of probesets was performed using “illuminaHumanv4.db” package [23].

### 2.2. Evaluation of Protein-Level Expression

Immunohistochemistry (IHC) data available in human protein atlas (https://www.proteinatlas.org, accessed on 1 August 2019) was used [24,25]. IHC stainings for proteins coded by 17 genes—ACSL4 (clone: HPA005552), RRM2 (clone: HPA056994), NEK2 (clone: CAB017530), KIF20A (clone: HPA036909), NCAPG (clone: HPA039613), TTK (clone: CAB013229), AKR1B10 (clone: HPA020280), PEG10 (clone: HPA029915), SULT1C2 (clone: HPA007190), COL15A1 (clone: HPA017913), CD24 (clone: CAB078471), SPP1 (clone: HPA027541), NQO1 (clone: HPA007308), PRC1 (clone: HPA034521), CCNB1 (clone: CAB003804), TOP2A (clone: HPA006458) and DTL (clone: HPA028016)—were evaluated in hepatocellular carcinomas and normal liver tissues (as control). Cholangiocarcinomas were not included in the assessments. A “percentage” and an “intensity” value were generated for each patient. Percentage was assessed as the percentage of the positive neoplastic cells (for HCC) and positive hepatocytes (for normal liver). Intensity was noted as the intensity of staining in positive neoplastic cells (for HCC) and positive hepatocytes (for normal liver) (1: mild, 2: moderate, 3: strong). A simplified H score was calculated by multiplication of percentage and intensity values. For tissues with duplicate cores, the scoring was performed based on evaluation of both cores generating an average score.

### 2.3. Bioinformatics Analyses

Hierarchical clustering was performed via Gene Cluster version 3.0 [26] using Euclidean distance as the similarity metric and complete linkage as the clustering method. For the validation dataset (GSE65359), K-means clustering was performed. Output visualization was conducted with Java Treeview [27]. In order to assess molecular differences, gene set enrichment analyses were performed by GSEA 4.0.3 software [28] using default settings, and gene sets were extracted from the Molecular Signatures Database (MSigDB)’s (https://www.gsea-msigdb.org/gsea/msigdb/index.jsp, accessed on 1 August 2019) c2 (c2.all.v7.2. symbols.gmt) and c5 (c5.all.v7.0.symbols.gmt) lists. Liver-cancer-related gene sets were extracted from the list of curated gene sets in MsigDB. Gene sets with a nominal *p*-value lower than 0.01 and a false discovery rate (FDR) below 0.25 were considered enriched. QuickGO [29] web-tool was used to understand functional patterns that are prominent among enriched gene sets obtained by GSEA. Functional annotation clustering analysis of gene lists was performed using DAVID [30,31]. Functional over-representation analyses for the proposed gene panel were performed using “enrichGO” function under “clusterProfiler” [32] package in R bioconductor and “Statistical overrepresentation test” module of PANTHER (Protein ANalysis THrough Evolutionary Relationships Classification System) [33,34]. CIBERSORT web tool (https://cibersort.stanford.edu/, accessed on 1 August 2019) absolute mode was used to estimate abundances of immune cell types [35]. Analyses were conducted with 100 permutations with default statistical parameters. Samples with a deconvolution *p* value higher than 0.05 were excluded from the analyses. Cell-cycle-related genes among the studied gene panel were extracted from the Reactome tool under “Cell Cycle (Homo sapiens)” (https://reactome.org/, accessed on 1 August 2019) [36]. The *p*-value adjustment was performed by the Benjamini-Hochberg method in R bioconductor using the *p*.adjust() function under the “stats” package. Volcano plots were generated via the Dash-Bio package for Python 3.8 [37]. Protein network analysis was performed using the QIAGEN Ingenuity Pathway Analysis (QIAGEN IPA) (QIAGEN Inc., https://digitalinsights.qiagen.com/IPA, accessed on 1 August 2019) application [38] based on log fold changes of 195 probesets between PCM1-U and PCM1-D groups in GSE83148. Protein–protein interaction (PPI) network was constructed via STRING (https://string-db.org/, accessed on 1 August 2019) at medium confidence (0.4) [39]. The network is restricted to physical interactions with experimental evidence. Maximum interactions for the first shell was set to “no more than 10 interactions”. Disconnected nodes were not included in the network.

### 2.4. Statistical Analyses

Coefficient of variation was calculated by division of standard deviation of each probeset to its mean. Student’s *t*-tests and Man–Whitney U tests were performed by Graphpad Prism 8.4.3.686 for Windows, GraphPad Software, San Diego, CA, USA, www.graphpad.com. IBM SPSS Statistics for Windows v.23 (IBM Corp., Armonk, NY, USA) was used for Chi Square and Fisher’s Exact tests. Benjamini-Hochberg method was used for *p* value adjustment. Gene expression and HCC prognosis relationships were tested using the coxph() function under the “survival” package in R bioconductor [40]. The *p* values below 0.05 were considered statistically significant in all comparisons.

## 3. Results

### 3.1. Identifying Transcriptomic Sub-Groups among CHB Patient Liver Samples

We hypothesized that variations in gene expression in the livers of CHB patients could be used to define sub-groups of patients with differential potential risk of disease progression. For this purpose, we analyzed 122 and 124 chronic HBV patients in the GSE83148 and GSE84044 datasets, respectively, and selected 500 probesets with the highest coefficient of variance independently for each dataset. We found that 90.8% of the probesets identified in the two lists were identical (476 of 500 probesets). Of these 476, we used those with annotated HUGO gene symbols in a hierarchical clustering analysis. Several clusters could be clearly identified for both datasets, from which we concentrated on those with more than 15 gene identifiers (six clusters in total). (Supplementary Figure S1, Supplementary Tables S7 and S9).

Clusters 1 to 6 included 14, 11, 18, 10, 52 and 176 unique genes, respectively (Supplementary Table S9). We performed functional annotation analysis of these genes via the DAVID tool within each cluster. We noted significant enrichments (Benjamini Hochberg corrected *p* value < 0.01) in Clusters 5 and 6. Cluster 1 included several pseudogenes and also genes related to neural processes such as ASTN2, SH3GL3 [41,42] and an olfactory receptor (OR7D2). Cluster 2 consisted of two genes related to neural functions, neural cell adhesion molecule 2 (NCAM2) and G Protein-Coupled Receptor 88 (GPR88) [43,44]. No strong enrichment patterns were noted in Cluster 3, which had two liver-enriched transcription factors, NR5A2 and FOXA1 [45,46]. Cluster 4 harbored several Y-chromosome-linked genes such as EIFAY, DDX3Y, USP9Y, TTTY14, TXLNGY, suggesting that this gene cluster identified genders. Additionally, “muscle protein”, “muscle filament sliding” and “troponin complex” were enriched for Gene Cluster 5, possibly reflecting metabolic stress and/or stromal cell presence [47,48]. However, in the 6th cluster, which had the highest number of genes, many proliferation-related gene sets, including “Cell cycle”, “cell division” and “mitosis”, suggestive of increased cell division and tumorigenesis, were significantly enriched. In addition, multiple gene sets related to the immune/inflammatory response to a virus, such as “killing of cells of other organism”, “immune response”, “Viral protein interaction with cytokine and cytokine receptor” and “Chemokine interleukin-8-like domain”, were also enriched, suggesting the presence of an anti-viral response. Based on these findings, we chose Cluster 6 for further analyses, as it contained functional groups that are likely to be related to carcinogenesis (Section 3.2), (Supplementary Figure S1). We named this signature PCM1, as explained in Section 3.2.

### 3.2. PCM1 Signature Identifies a “Proliferation, Cancer and M1 Macrophage” Related Phenotype

**Figure 1:**
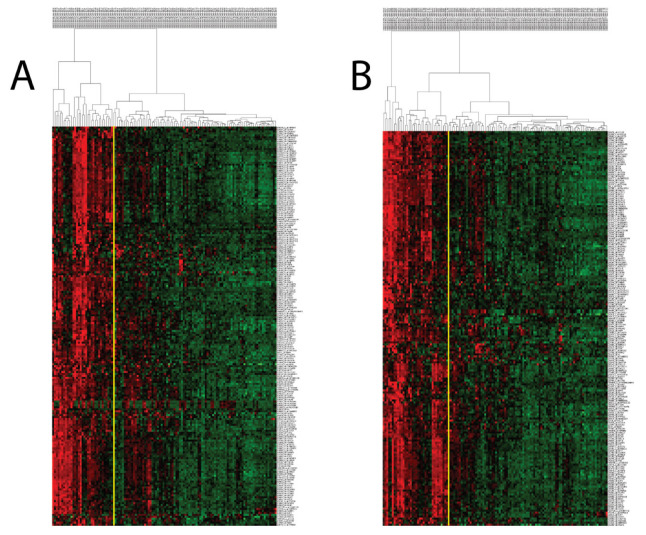
Hierarchical clustering of CHB livers in GSE83148 (**A**) and GSE84044 (**B**) with PCM1 probesets reveals the presence of two distinct sample groups. Expression values of 204 and 197 probesets (rows) identified in Supplementary Figure S1 (Cluster 6) were used to analyze CHB livers (columns), showing the two sample groups (PCM1-U and PCM1-D). Red, black and green colors indicate high, moderate and low expression, respectively. The yellow vertical line indicates the demarcation of the two sample clusters.

When we performed hierarchical clustering with PCM1 genes (probesets) in the two discovery datasets (GSE83148: 204 probesets/183 genes, GSE84044: 197 probesets/177 genes), we observed two distinct patient groups that were identified based on whether the samples had a high or low expression of these genes. PCM1 genes identified using the two datasets were highly similar, with 195 probesets/176 genes in common (Supplementary Figure S2, Supplementary Table S7). Patients with upregulated PCM1 gene expression comprised 27% and 29% of the samples in GSE83148 and GSE84044, respectively (Figure 1). To test the robustness of PCM1, we then used these 195 probesets for the k-means clustering (k = 2) of a third dataset (GSE65359) and again observed clear sub-groups with high and low expression consisting of 29 and 54 CHB liver samples, respectively (Supplementary Figure S3A). An independent validation of PCM1 was performed in the GSE83898 dataset, which consisted of formalin fixed paraffin embedded (FFPE) biopsies collected from the livers of chronic HBV patients (n = 73). A total of 167 PCM1 genes were available in this dataset. Although this dataset harbored differences in terms of sample type (FFPE), microarray platform (Illumina) and PCM1 gene number compared to aforementioned datasets, we were able to clearly identify two major groups with similar expression profiles, as we had with other datasets (Supplementary Figure S3B). Thus, PCM1 could consistently identify two main groups of CHB liver samples in which all the genes of PCM1 were either up- (PCM1-U) or down-regulated (PCM1-D).

To better understand the molecular differences between the sub-groups identified by PCM1, we performed GSEA analyses using the GSE83148 and GSE84044 datasets. A total of 574 and 730 gene sets were enriched in PCM1-U samples in the GSE83148 and GSE84044 datasets, respectively, of which 500 were common to both (Supplementary Table S6). Among PCM1-D samples, we could not find common gene sets that were significantly enriched, which likely indicates that this is still a heterogeneous population. This is also evident in terms of the clinical characteristics of this group, as shown in Tables 1 and 3 (see Section 3.6). We therefore performed a series of GSEA analyses within PCM1-D samples grouped according to different clinical characteristics (ALT, AST, or HBV DNA levels). However, GSEA of such subgroups still did not reveal consistently enriched gene sets (data not shown). However, the PCM1-D group was shown to have a distinct and common immune phenotype (see Section 3.5).

**Figure 2:**
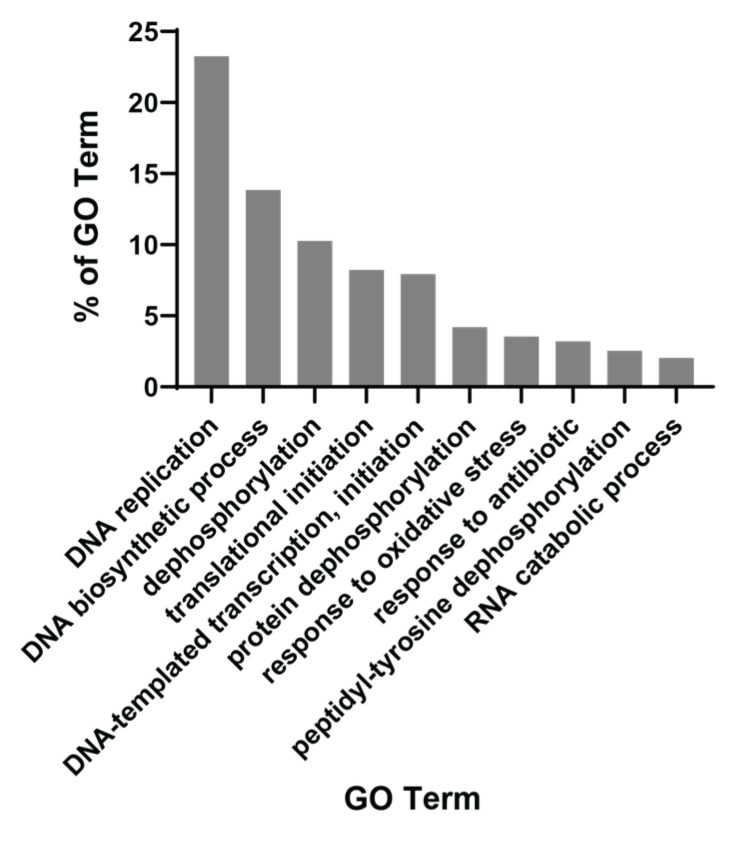
QuickGO-based GO term annotation for commonly enriched gene sets (Biological Process) in PCM1-U group.

We then used the QuickGO tool to obtain gene ontology patterns prominent among 500 commonly enriched gene sets in PCM1-U samples. This showed that the foremost biological mechanisms were “DNA replication” and “DNA biosynthetic process”, included in almost 40% of all enriched gene sets (Figure 2). Other mechanisms included within >5% and ≤10.3% of the gene sets were “dephosphorylation”, “translational initiation” and “DNA-templated transcription, initiation”. These findings suggest a replicative and transcriptionally active profile. More specifically, we observed a change in metabolism of various biological building blocks (nucleotides, lipids, proteins, carbohydrates (aminoglycans)), suggesting that all macromolecules are being synthesized more in PCM1-U, which is in line with a proliferative profile, since these molecules are required for the generation of new cells. Multiple gene sets related to “ROS metabolic process” were enriched in this group, which is a hallmark of chronic HBV infection and advanced liver disease [49]. Among the enriched gene sets, WNT and ERK signaling were noted, which are among the important players of HCC carcinogenesis [50]. Overall, the PCM1 signature identified a subgroup among HBV-infected livers with elevated cellular proliferation and biosynthesis with the activation of HCC-specific molecular pathways.

In addition to these, enhanced IL-1 production (“Interleukin 1 production”, “Interleukin 1 beta production”), secretion (“Interleukin 1 secretion”) and signaling (“Interleukin 1 mediated signaling pathway”, “Response to interleukin 1”) was evident in these analyses for the PCM1-U group (Supplementary Table S6). The gene sets named “Neutrophil migration”, “Regulation of leukocyte mediated immunity”, “Leukocyte migration”, “Myeloid leukocyte differentiation” and “Leukocyte chemotaxis” as well as “Macrophage activation” and “Interleukin-8 production” were also enriched in PCM1-U samples. Moreover, the enrichment of inflammatory mechanisms such as tumor necrosis factor (TNF), nuclear factor kappa B (NF-κB) and tumor growth factor beta (TGFβ) signaling pathways was noted. Other enriched GO terms included “Innate immune response activating cell surface receptor signaling pathway”, “Regulation of viral transcription” and “Response to virus”, which altogether supported the enrichment of a clear innate immune response to viral infection and inflammatory activity in PCM1-U. Therefore, inflammation, in response to the viral infection, is markedly enhanced in the PCM1-U group.

### 3.3. Transcriptomic Changes Suggest a Higher Risk of Malignant Transformation in PCM1-U Group

In order to evaluate the activation of HCC-related molecular mechanisms in PCM1 classified groups, we performed GSEA analysis for curated liver-cancer-related gene sets from the MsigDB database in GSE83148 and GSE84044. A total of 17 such gene sets, listed in Supplementary Table S4, were enriched in PCM1-U samples in both datasets. Enrichment plots of four (out of seventeen) representative gene sets are shown in Supplementary Figure S6. The “Boyault Liver Cancer Subclass G3” gene set includes genes that are upregulated in a liver cancer subclass which is characterized by mutations of p53 and the overexpression of genes controlling the cell cycle [51]. In this line, we observed the enrichment of the “Chiang Liver Cancer Subclass Proliferation Up” gene set in PCM1-U, which contains genes up-regulated in the ‘proliferation’ subclass of HCC [52]. Another enriched gene set was the “Hoshida Liver Cancer Subclass S1”, which includes genes that are upregulated in liver tumors with aberrant activation of the WNT signaling pathway [53]. In addition, enrichment of the “Lee Liver Cancer Survival DN” gene set, which harbors genes that are associated with poor survival in HCC, was noted [54]. Altogether, these data indicate that PCM1-U samples have a transcriptomic profile similar to tumors that harbor p53 mutations, have active WNT signaling and are proliferative, and thus show a potential for rapid progression.

As enrichment of liver-cancer-related gene sets suggested that a subset of PCM1 genes may also have roles in HCC tumorigenesis, we compared the expression of PCM1 genes between HCC tumors and normal liver. For this purpose, we used GSE121248 and GSE14520 datasets containing both normal and tumor samples. These datasets included 176 and 132 PCM1 genes (434 and 236 probesets), respectively. Among 54 and 28 unique genes that were differentially expressed between tumor and normal (Benjamini Hoechberg adjusted *p* < 0.05, absolute log fold change > 1.5), 92.6% and 85.7% were expressed at higher levels in PCM1-U samples for GSE121248 and GSE14520, respectively (Supplementary Figure S7). The full list of the differentially expressed genes is given in Supplementary Table S8. Overall, these results show that PCM1-U marks a proliferative subgroup among livers with CHB, harboring malignant transcriptomic patterns.

### 3.4. The Majority of PCM1 Genes Are Related to Mitosis and Immune Response

To better characterize the PCM1-defined sub-groups, we performed over-representation analysis with the 176 genes using the PANTHER knowledgebase and enrichGO function in R. The top 20 most significant mechanisms identified by enrichGO were all cell-cycle-related, confirming findings we previously obtained by QuickGO (Supplementary Figure S4). PANTHER analysis revealed significant over-representation of “regulation of mitotic metaphase/anaphase transition”, “mitotic spindle organization (GO:0007052)”, “mitotic sister chromatid segregation (GO:0000070)”, “cell cycle G1/S phase transition (GO:0044843)”, “mitotic prometaphase (GO:0000236)”, “mitotic cell cycle phase transition (GO:0044772)”, “cell division (GO:0051301)” and “regulation of cell adhesion (GO:0030155)” among the top mechanisms when sorted by fold enrichment. To further support the clear mitosis-related pattern observed in these analyses, we analyzed the expression of PCM1 genes that are associated with cell cycle in the Reactome database (CENPK, MND1, CDC6, NCAPG, RRM2, CENPU, MAD2L1, NEK2, NDC80, NUF2, KIF20A, BUB1, SPC25, MCM10, CCNB1, CCNB2, PHLDA1, CCNA2). All 18 genes tested were expressed at a significantly higher level in PCM1-U compared to PCM1-D samples in GSE83148 (Student’s *t*-test, adjusted *p* < 0.0001) (Supplementary Figure S5). Several immune-system-related gene sets were also featured such as “chemokine-mediated signaling pathway (GO:0070098)”, “neutrophil chemotaxis (GO:0030593)”, “killing of cells of other organisms (GO:0031640)”, “antimicrobial humoral immune response mediated by antimicrobial peptide (GO:0061844)” and “response to bacterium (GO:0009617)” (Supplementary Table S2).

In order to identify the molecular networks associated with PCM1 gene expression changes in CHB liver, we used QIAGEN Ingenuity Pathway Analysis. The top three networks with the highest number of focus molecules are shown in Supplementary Figure S8. The highest-scoring network (Nr. 1) has NF-κB (a pivotal mediator of inflammatory responses) as a hub molecule linked to the upregulation of numerous PCM1 molecules (Supplementary Figure S8A), in line with our earlier results showing enrichment of NF-κB in the GSEA analysis (see Section 3.2). In addition, an upregulation of multiple cell-cycle-related genes (CCNB1, E2F7, ECT2, CDC6, E2F8, HELLS, CCNA2) revealed a sub-network within Network 1, which was associated with the predicted inhibition of RB transcriptional corepressor 1, an important regulator of cell cycle which acts as a growth suppressor [55]. This data suggested a higher rate of cell cycle activity via the suppression of Rb in PCM1-U tissues associated with inflammation. The second network with the highest score (Network 2) had CXCL8 “C-X-C Motif Chemokine Ligand 8” as a hub PCM1 gene, which is involved in inflammatory processes and is aberrantly regulated in many inflammatory-mediated diseases [56]. This network predicts the activation of TCR “T cell receptor”, which leads to the activation of CTLA4 “Cytotoxic T-Lymphocyte Associated Protein 4”, CXCL8, CD69 “Early T-Cell Activation Antigen P60”, SOCS3 “Suppressor of Cytokine Signaling 3”, RGCC “Regulator of Cell Cycle” (PCM1 genes) and CD3, Jnk and Akt complexes, highlighting the T-cell presence in PCM1-U samples (Supplementary Figure S8B). The third network predicted the activation of multiple pathways related to cellular growth, such as the MAP2K1/2, ERK1/2 and PI3K complex (Supplementary Figure S8C). The activation of P38 MAPK is involved in the regulation of the synthesis of inflammatory mediators and thus is a potential target for anti-inflammatory therapeutics [57].

In order to evaluate protein–protein interactions that potentially underlie the biological enrichments observed in our data, we constructed a protein–protein interaction network (PPI) via STRING (https://string-db.org/, accessed on 1 August 2019) based on 176 PCM1 genes that showed a significant PPI enrichment (*p* < 1 × 10^−16^) (Supplementary Figure S9). Major hubs in the network CDK1, BUB, CDC6 revealed protein–protein interactions with cell-cycle-related genes.

Overall, network analyses strongly supported our previous findings regarding a pro-inflammatory and actively proliferating profile for PCM1-U, and predicted the inhibition of a known tumor suppressor that was often inactivated in HCC.

Collectively, as shown via multiple functional evaluation methods, our findings clearly indicated higher proliferative and inflammatory activity in PCM1-U liver samples among HBV patients. As we also noted immune-system related functional enrichments in addition to an active cell cycle within PCM1 (Supplementary Table S2), we next aimed to determine whether the presence of specific immune cell types in the PCM1 defined groups differ, using transcriptomic data.

### 3.5. Specific Immune Cell Types Are Prominent in PCM1 Groups

**Figure 3:**
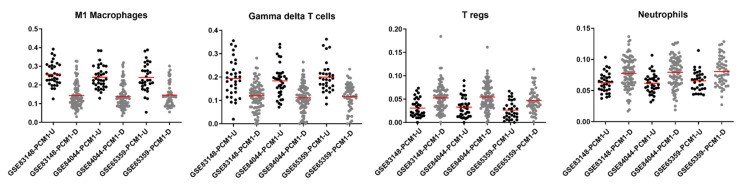
CIBERSORT fractions of M1 macrophages, gamma delta T cells, regulatory T cells and neutrophils within PCM1-U and -D groups in GSE83148, GSE84044 and GSE65359. Red horizontal line shows the mean value of fractions. Y axis: fraction of immune cells.

Inflammation is known to play a vital role in HCC development and progression [58]. To understand the nature of an immune involvement within PCM1-classified samples in the GSE83148, GSE84044 and GSE65359 datasets, we utilized the CIBERSORT web tool, which allows immune cell profiling by the deconvolution of microarray data [59]. Among the twenty-two immune cell types included in this approach, seven cell types that were absent in more than 50% of the samples were excluded from further analyses. Fractions of the remaining 15 immune cell types were then compared between the PCM1-U and -D samples. When CIBERSORT-based adjusted *p* values were ranked from small to large in all three datasets, the top four significant cell types based on sum of ranks included M1 macrophages, gamma delta T cells, regulatory T cells (Tregs) and neutrophils (Supplementary Table S3). The fraction of M1 macrophages and gamma delta T cells were significantly higher in PCM1-U, while Tregs and neutrophils were significantly higher in PCM1-D CHB liver samples (Supplementary Table S3, Figure 3). The absolute fold difference between abundances was most prominent for M1 macrophages (around 0.1 for all datasets), whereas other cell types with significant *p* values showed fold differences between 0.01 and 0.07 (Supplementary Table S3). M1 macrophages are well known pro-inflammatory cells [60], suggesting a more inflammatory phenotype in PCM1-U samples. Gamma delta T cells, on the other hand, have been shown to have both anti- as well as pro-tumor inducing capabilities in chronic liver disease [61,62]. Regulatory T cells, which were higher in PCM1-D, have a role in the maintenance of the immunosuppressive microenvironment in the liver and are also involved in the development of cirrhosis, the transformation of cirrhosis to HCC, and the progression of HCC [63]. Since neutrophils are crucial in the initiation of inflammation, altogether these data suggest that PCM1-U and PCM1-D groups have different immune activity, a more inflammatory microenvironment for PCM1-U, and a rather immunosuppressive environment for PCM1-D.

### 3.6. PCM1 Groups Show Differences in ALT, AST, Viral Phase, Level of Fibrosis and Inflammation

Currently, biomarkers such as ALT, AST and HBV DNA are used to determine clinical disease progression in patients with CHB. Higher levels or all three biomarkers predict an unfavorable prognosis. We evaluated whether patients assigned to the PCM1 subgroups (Figure 1) differed in terms of these parameters, using GSE83148 and GSE84044. We observed that HBV DNA levels were not associated with PCM1 clustering (Table 1). However, almost all PCM1-U samples also had high AST and ALT levels (Table 1). On the other hand, among the samples with a PCM1-D profile, all three clinical parameters evaluated were almost randomly distributed (Table 1), which was in line with the heterogeneous profile of these patients in GSEA analysis. Statistically, PCM1-U and PCM1-D differed significantly in terms of the distribution of ALT and AST levels, but not HBV DNA levels (Table 2).

**Figure 4:**
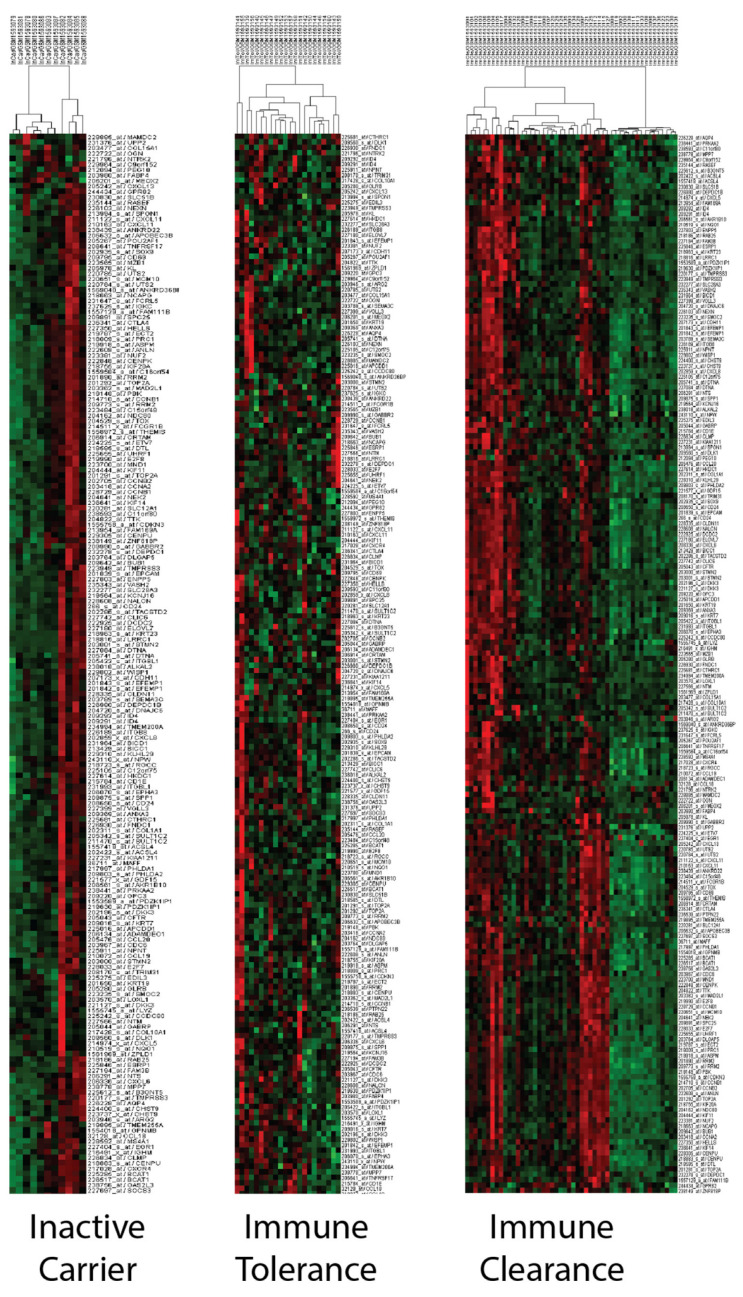
PCM1-based sub-groups can be identified within inactive-carrier and immune-clearance phases but not in immune tolerance. Hierarchical clustering was performed with 195 probesets/176 genes separately in samples at inactive-carrier, immune-tolerance and immune-clearance phases in GSE65359.

ALT, AST and HBV DNA levels are known to vary during the phases of the virus infection [64,65]. Therefore, we investigated the relationship between these phases and the PCM1 signature in silico. The current terminology for viral phases (HBeAg-positive chronic infection, HBeAg-positive chronic hepatitis, HBeAg-negative chronic infection and HBeAg-negative chronic hepatitis [66]) differ from those used in this study (inactive carrier, immune tolerance, and immune clearance) due to the availability of the former terminology in the microarray datasets. For this purpose, we used the GSE65359 dataset, which includes viral phase data from 83 CHB liver samples. The PCM1-U and -D samples were clearly defined in this dataset, as shown in Supplementary Figure S3. The viral phase distribution among the PCM1-U and -D samples is shown in Table 3. All patients within the immune-tolerant phase were in the PCM1-D group, which could be expected, as these patients have minimal inflammation and fibrosis and low ALT activity [67]. In contrast, the majority of the PCM1-U samples (86.2%) were in the immune-clearance phase, where chronic active inflammation is observed, indicating a worse predicted outcome. In addition, we observed an equal number of PCM1-U and PCM1-D samples within the immune clearance phase. During the inactive carrier phase, where patients have mild hepatitis and minimal fibrosis, we observed about half of the samples identified as either PCM1-U or -D. This suggests that the PCM1 classification might be an informative tool for CHB cases, especially when the patients are in immune-clearance or inactive-carrier phases. The clustering analyses with 195 probesets/176 genes performed in each phase separately showed the effectiveness of PCM1 clustering in the “immune clearance” and “inactive carrier” phases but not in the “immune tolerance” phase in GSE65359 (Figure 4). To evaluate the relationship between PCM1 and the Scheuer Scores of the samples, we analyzed the GSE84044 dataset [68]. This score indicates necroinflammatory activity and fibrosis in chronic hepatitis B. While 94.3% of the samples in the PCM1-D group had Scheuer scores below or equal to two for necroinflammatory activity, 83.3% of the samples in the PCM1-U group had grades of two and higher (*p* < 0.001, Supplementary Table S1). A similar pattern was observed for the Scheuer score of fibrosis and cirrhosis; 92.1% of the samples in PCM1-D samples were of grade 0–2, whereas 91.6% of the samples in PCM1-U had grades 2–4 (*p* < 0.001, Supplementary Table S1). These data show that the PCM1 classification is significantly related to the level of fibrosis and inflammation in the sampled tissues.

### 3.7. PCM1 Genes Are Associated with Clinical Outcome in HCC

To assess the prognostic association of the individual genes specifically in HCC patients (HBV+), we applied cox regression analyses for 110 out of the 195 probesets available in GSE14520. When the expression values were used as continuous variables, the expression of twenty-two probesets (nineteen genes) was associated significantly with overall survival (cox *p* < 0.05), and twenty-one of them had a hazard ratio above one (Supplementary Table S5). Eight probesets (seven genes), which had *p* values lower than 0.01, were all associated with shorter overall survival (Supplementary Table S5). When the prognostic relationships were evaluated categorically (groups with high and low expression were compared) at all possible cut-offs, 77 out of 110 probesets were significantly associated with overall survival at one or more cut-offs (data not shown). Therefore, in addition to the relationship of this gene panel with the development of HCC in HBV-infected patients, higher expression of a subset of these genes is also related to poor disease progression in HCC.

### 3.8. PCM1 Genes Validated by Immunohistochemistry in Hepatocellular Cancer Samples

**Figure 5:**
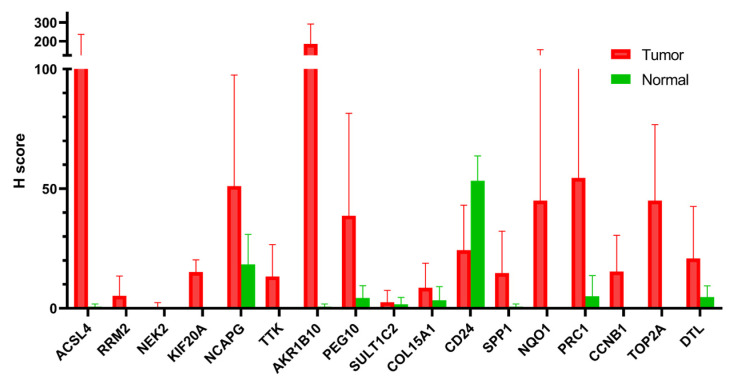
Immunohistochemistry-based evaluation of proteins coded by 17 PCM1 genes. Mean H scores are shown in the y-axis. Error bars indicate standard deviations.

To validate the potential link between PCM1 and malignant transformation in CHB liver, we investigated the protein level expression of these genes in Human Protein Atlas (https://www.proteinatlas.org/, accessed on 1 August 2019). Among the differentially expressed PCM1 genes between liver tumors and normal liver tissues at the mRNA level, we noted twenty-three common PCM1 genes between the two lists (Supplementary Table S8). The immunohistochemistry (IHC)-based staining images of 17 out of 23 genes, available in Human Protein Atlas, were included in the analysis. Based on the percentage of the positive neoplastic cells and the staining intensity (Supplementary Table S10), a simplified H-score was calculated as a measure of gene expression at the protein level (see Section 2.2). Fifteen of the seventeen genes had an H score of less than five in normal liver, indicating minimal or no protein-level expression. In contrast, as shown in Figure 5, all genes except for CD24 have a higher mean H score in hepatocellular tumors compared to normal liver. We noted a dramatically high mean H score for ACSL4 and AKR1B10 in tumors compared to normal liver (mean tumor H score minus mean normal H score were 105.5 and 185.0 respectively). Taking into account the fact that IHC stainings were obtained from an independent cohort and that the method measures gene expression at the protein level, we believe this data very strongly supports our in silico data supporting the strength of PCM1 genes as predictors of liver cancer development in CHB.

## 4. Discussion

HBV-associated HCC development is a complex process that includes deregulation in multiple cellular signaling pathways. In response to the degradation products of apoptotic cells and the viral antigens in the microenvironment of HBV-infected liver, inflammatory and cytokine reaction is driven by immune cells [69,70]. As a consequence of this biological complexity, detecting the CHB patients who are more likely to develop HCC can be challenging. There are several multi-parameter-based methods developed for the assessment of HCC development risk [71,72,73], but in clinical practice, treatment decisions are mainly guided by the serial testing of ALT and HBV DNA levels [7]. In our study, transcriptomic data from HBV-infected livers were utilized to define a signature that could predict patients with higher risk of developing HCC.

Based on a list of 176 genes, which we called “PCM1”, two main transcriptomic sub-groups were identified among patients with CHB, consistently in three datasets. The patients in the “PCM1-U” group mainly had high ALT and AST levels; however, the patients in the “PCM1-D” group were more heterogeneous, including patients with various levels of HBV DNA and ALT and AST. High ALT and AST levels are considered as signs of acute and chronic hepatocyte cytotoxicity, showing that the PCM1-U group almost homogeneously had a higher level of liver damage in addition to higher fibrosis and inflammation. Our results showed that cell-cycle-related gene expression was dramatically higher, and multiple curated liver-cancer-related gene sets were enriched clearly in the “PCM1-U” group. In line with this, ERK and WNT signaling-related gene sets were enriched in this group, both of which are among the major players of HCC carcinogenesis [50]. These findings were also supported by IPA network analysis-based prediction of ERK1/2 activation. ERK can be activated both through growth factor receptors and HBV infection, triggering the activation of downstream effectors and resulting in the transcription of genes that drive cell proliferation [50]. The WNT pathway is activated in around 50% of HCC tissues regulating multiple cellular processes related to the initiation, growth, survival, migration, differentiation and apoptosis of HCC [74]. Our finding that the majority of the differentially expressed PCM1 genes between HCC tumor and normal are higher in HCC tumors also suggests that the PCM1 signature identifies HCC-specific expression patterns. The IPA analysis of the molecular networks revealed the activation of cell growth pathways (MAP2K1/2, ERK1/2 and PI3K complex) and the inhibition of tumor suppressor protein Rb, in addition to cell-cycle-related protein–protein interactions involved in this signature, support these findings at also other biological levels other than RNA. Overall, these results suggest that the “PCM1-U” group may represent a sub-group of HBV-infected livers which activated molecular mechanisms pointing to an early stage of carcinogenesis and thus harbor a higher risk for HCC development.

As guidelines suggest treatment for patients with high HBV DNA and ALT levels [7], the presence of such patients in both PCM1-U and PCM1-D groups may indicate that there are patients with distinct transcriptomic profiles among patients who are treated according to the same protocol. Therefore, the transcriptomic features defined in this study may provide the basis for the enlightenment of molecular differences among CHB patients that may contribute to personalized medicine in the future.

Chronic HBV infection progresses through four phases, which are assessed mainly by the presence of HBeAg, HBV DNA levels, alanine aminotransferase (ALT) values, and the presence or absence of liver inflammation [75]. When we evaluated the overlap of the PCM1 signature with the viral phases in one dataset that had viral-phase data, we observed that the immune-tolerant phase CHB livers were assigned to the PCM1-D group, whereas other phases included both PCM1-U and PCM1-D samples. PCM1 subgroups can be assigned within samples at the immune-clearance phase, which are known with the activation of HBeAg-specific T cell clones, inflammation [75], liver fibrosis, and inactive carriers, which constitute the largest group of CHB patients with normal ALT levels and minimal or no necroinflammation [76]. Successful immune activity in the immune-clearance phase results in HbeAg loss, the development of anti-Hbe antibodies and thus a transition to the inactive carrier state; whereas other patients who have no serologic clearance may have liver damage, which may lead to fibrosis, cirrhosis and a higher risk of HCC [67]. Therefore, our classification may be a potential transcriptomic indicator of how this progression will take place, suggesting that there is a “PCM1-D” sub-group with a low risk of malignant transformation among patients at the immune-clearance phase. Furthermore, a different follow-up schedule for patients at the inactive state with a “PCM1-U” profile who may be at risk of re-activation can be suggested in the long term, since 20–30% of those patients can undergo spontaneous re-activation [76]. In summary, our findings suggest that patients with livers at immune-clearance and inactive-carrier phases are heterogeneous and can be sub-divided into PCM1 groups based on their transcriptomic profiles.

The comparison of specific immune cell population fractions in the microenvironment showed that M1 macrophage fractions are higher in the PCM1-U group than in the PCM1-D group. As HCC is well known as an inflammation-linked cancer [58] and M1 Macrophage phenotype is the classically activated macrophage which exerts pro-inflammatory properties [77], a more inflammatory state identified in the PCM1-U group may contribute to a higher HCC progression risk. In this line, we observe an enrichment of macrophage-activation-related gene sets in this group based on GSEA analysis. Our results showed a consistent enrichment of gene sets related to proinflammatory IL-1 secretion, the IL-1 signaling pathway, and IL-8 production in the PCM1-U group. IL-8 is well-known for its function to induce neutrophil migration to the inflammatory environment, forming the “cellular first line of defense”, and is also widely used as a diagnostic and prognostic marker for infectious and inflammatory conditions [78]. These data, together with the enrichment of the gene sets related to a higher leukocyte migration and an active leukocyte-mediated immunity in the PCM1-U samples, clearly support a stronger inflammatory response in this group. These transcriptome-based findings are in line with the histological assessments, as the Scheuer score of both necroinflammation and fibrosis were dramatically higher in the PCM1-U samples. TGFβ and NF-κB signaling pathways were among the enriched processes in PCM1-U. In line with this, NF-κB was a hub molecule with many associations in the IPA network. Pro-inflammatory NF-κB signaling was shown to be activated by HBx and several other HBV related mechanisms, such as ROS, which was also found as enriched in PCM1-U [79]. Oxidative stress induced by hepatitis viruses is considered as one of the driving factors of neoplastic transformation in the liver [49], supporting our findings regarding a higher risk for HCC in the PCM1-U group.

HBV infection has been reported to be involved in the derangement of many metabolic pathways including glucose, lipid, nucleic acid, bile acid and vitamins, which may also contribute to pathological processes such as the development of HCC [80]. In our data, we found gene sets related to the regulation of lipid, protein, nucleotide and carbohydrate metabolisms enriched in PCM1-U, suggesting that these processes were rather more active in PCM1-U compared to PCM1-D. These metabolic changes are likely to be linked with a higher proliferative rate in this group, since a higher amount of biological building blocks are needed for new cells to be generated.

The immunotolerance phase is known as an early phase of CHB infection associated with high HBV replication and a lack of clinical signs of inflammation [81]; Tregs are one of the main players in the inhibition of the antiviral activity of effector T cells and reducing immunopathological liver damage [63]. In our study, we found that Tregs were higher in the PCM1-D group compared to PCM1-U, which makes sense since M1 macrophage and T cell fractions were also significantly lower in the PCM1-D group. Altogether, these results clearly show an immunosuppressive phenotype for the PCM1-D group.

Gamma delta T cells are known to have various and even contradictory roles during HBV and HCV infections [82]. While IFN-γ- or TNF-α-producing γδ T cells can inhibit acute and chronic HBV infection, human CD4-CD8-γδ T cells and mouse IL-17-producing Vγ4 T cell subsets contribute to the progression of chronic HBV infection. As we found a higher level of gamma delta T cells in the PCM1-U group that had more inflammation and cellular proliferation and thus a likely profile to progress, the gamma delta T cells analyzed may likely be of the CD4-CD8 type. Edwards et al. showed that a T cell subgroup that coexpresses αβ and γδ TCRs was associated with a hyper-inflammatory phenotype and that they secrete IFN-γ, IL-17 and GM-CSF upon antigen stimulation [83]. Thus, a higher fraction of γδ T cells in the PCM1-U group which had more inflammation may be in line with these findings. Peripheral blood T cell gamma delta cells are known to be decreased in CHB patients, and the level of these cells is negatively correlated with ALT and AST levels [84]. In our analyses, gamma delta T cells were more abundant in the liver samples of the PCM1-U group which had high ALT AST levels. Enhanced gamma delta T cell function has been suggested to contribute to a better IFN-α treatment response, as higher levels of gamma delta T cells are found in responders [85]. Therefore, the increase in gamma delta T cells in the PCM1-U group may suggest that this group may have a higher potential to be a candidate for IFN-α treatment.

This study evaluated the transcriptomic variations among livers with CHB that also relate to clinical parameters and disease progression. Due to the lack of HBe antigen level and HCC development information in the datasets, these data could not be directly related to our main findings, which can be considered as a limitation of this study. However, we showed that proteins encoded by PCM1 genes that showed significantly higher transcription levels in liver tumors compared to normal tissue were primarily stained positive in liver tumors in an independent cohort, while minimal or no staining was observed in normal liver, validating the potential of these markers as cancer predictors. Among the PCM1 genes, AKRB10 showed the highest difference between tumor and normal, with a mean H score in normal smaller than one, while mean the H score in tumors was 185.8. This gene was implicated in hepatocarcinogenesis via modulation of proliferation and apoptosis [86]. Another study suggested the cell-survival-promoting role of AKR1B10 is dependent on its role in lipid synthesis and eliminating carbonyls [87]. AKR1B10 was previously listed in an HCC-specific gene list [88,89]. The IHC-based assessment of AKR1B10 in patients with chronic HCV and HBV infection showed that high AKR1B10 expression was an independent risk factor for HCC (HRs: 6.43 and 10.8, *p* < 0.001, respectively). Five-year cumulative incidences of HCC with high and low AKR1B10 expression were 22.8% and 2.2% in chronic HCV and 20.6% and 2.6% in chronic HBV, respectively (*p* < 0.001) [90,91]. Serum AKR1B10 has been suggested as a biomarker for early stage HBV/HCV-related HCC [92]. These findings confirm that our method indeed identifies genes that are likely to be markers of liver carcinogenesis. ACSL4 “acyl-CoA synthetase long-chain family member-4”, which was involved in the hormonal regulation of steroidogenesis, had the second-highest mean tumor H score in our study [93]. Previous studies suggested a role for ACSL4 in a non-apoptotic cell death mechanism, ferroptosis, in several liver diseases including viral hepatitis [94]. To our knowledge, our study is the first to show this gene’s putative role as an HCC marker for patients with CHB.

Future studies with a group of HBV + CHB liver samples from patients with and without known HCC development would be valuable for testing the potential clinical use of genes identified within this study.

## 5. Conclusions

Here, we identified a novel gene panel, PCM1, that can be utilized to evaluate cancer-related expression patterns in HBV-infected livers. Bioinformatics-based analyses showed that PCM1-U samples harbored higher inflammatory and proliferative activity, suggesting a higher likelihood of malignant transformation in the liver. A subset of PCM1 genes validated at the protein level could generate a basis for further smaller gene signatures that can potentially be useful in clinics upon validation in other cohorts.

## Figures and Tables

**Table 1:** Clinical characteristics of PCM1-U and PCM1-D patients.

**PCM1-U**	**ALT (IU/L)**					
**AST**	**≤40**		**>40**	**HBVDNA**		
**(IU/L)**	**GSE83148**	**GSE84044**	**GSE83148**		**GSE84044**	**(Copies/mL)**
**≤35**	0 *	0	0		0	**≤1 × 10^6^**
**>35**	0	0	14		15	**≤1 × 10^6^**
**≤35**	0	0	0		1	**>1 × 10^6^**
**>35**	0	1	15		16	**>1 × 10^6^**
**PCM1-D**	**ALT (IU/L)**					
**AST**	**≤40**		**>40**	**HBVDNA**		
	**GSE83148**	**GSE84044**	**GSE83148**		**GSE84044**	**(Copies/mL)**
**≤35**	8 *	14	1		4	**≤1 × 10^6^**
**>35**	2	1	7		11	**≤1 × 10^6^**
**≤35**	13	16	4		3	**>1 × 10^6^**
**>35**	2	2	18		14	**>1 × 10^6^**

* The numbers of patients with specified AST, ALT and HBV DNA levels are shown.

**Table 2:** Association of PCM1 with ALT, AST and HBV DNA in GSE83148 and GSE84044.

**GSE83148**						
	**PCM1-U**		**PCM1-D**		**X^2^**	*p* Value *
**Clinical Data**	n **	% **	n **	% **		
**ALT** (IU/L):						
≤40	1	3.30%	37	49.30%	19.635 ^a^	<0.001
>40	29	96.70%	38	50.70%		
**AST** (IU/L):						
≤35	1	3.30%	41	54.70%	23.528 ^b^	<0.001
>35	29	96.70%	34	45.30%		
**HBV DNA** (copies/mL):						
**≤1 × 10^6^** **>1 × 10^6^**	14 16	46.70% 53.30%	22 38	36.70% 63.30%	0.833 ^c^	0.494
**GSE84044**						
	**PCM1-U**		**PCM1-D**		**X^2^**	*p* Value *
**Clinical Data**	n **	% **	n **	% **		
**ALT** (IU/L):						
≤40	1	3.00%	39	53.40%	24.564 ^d^	<0.001
>40	32	97.00%	34	46.60%		
**AST** (IU/L):						
≤35	1	3.00%	43	59.70%	29.874 ^e^	<0.001
>35	32	97.00%	29	40.30%		
**HBV DNA** (copies/mL):						
**≤1 × 10^6^** **>1 × 10^6^**	16 19	45.70% 54.30%	37 42	46.80% 53.20%	0.012 ^f^	1.000

^a^ 0 cells have expected count less than five. The minimum expected count is 10.86. ^b^ 0 cells have expected count less than five. The minimum expected count is 12.00. ^c^ 0 cells have expected count less than five. The minimum expected count is 12.00. ^d^ 0 cells have expected count less than five. The minimum expected count is 12.45. ^e^ 0 cells have expected count less than five. The minimum expected count is 13.83. ^f^ 0 cells have expected count less than five. The minimum expected count is 16.27. * Chi-square ** Numbers and percentages of patients with the specified clinical parameters.

**Table 3:** Distribution of viral phases within PCM1-U and PCM1-D groups in GSE65359.

**Viral Phase**	**PCM1-U**		**PCM1-D**		**Chi-Square**
	n *	% *	n *	% *	X^2^ = 16.8135, *p* < 0.001
Immune Tolerance	0	0.00%	22	40.70%	
Immune Clearance	25	86.20%	25	46.30%	
Inactive Carrier	4	13.80%	7	13.00%	
Total	29	100%	54	100%	

* Numbers and percentages of patients with the specified clinical parameters.

## Data Availability

Transcriptomic data used in this study was not produced by the authors; instead, our study included the analysis of previously reported microarray data. Publicly available datasets (GSE83148, GSE84044, GSE65359, GSE14520, GSE121248, GSE83898) used in this study can be obtained from NCBI GEO platform (accessed on 1 August 2019) (https://www.ncbi.nlm.nih.gov/geo/query/acc.cgi).

## Supplementary Materials

The following supporting information can be downloaded at: www.mdpi.com/article/10.3390/biology12020188/s1, Figure S1: Hierarchical clustering of CHB liver samples with the common most variant probesets in GSE83148 (a), GSE84044 (b); Figure S2: Venn diagrams showing overlap of probesets (a) and genes (b) identified based on independent datasets (PCM1); Figure S3: Hierarchical clustering of liver samples from CHB patients in GSE65359 and GSE83898; Figure S4: Functional over-representation of 176 gene panel; Figure S5: Cell cycle related gene expression in PCM1-U (red) and PCM1-D (green) groups (GSE83148); Figure S6: Enrichment plots of the curated liver cancer related gene sets in GSE83148 (a) and GSE84044 (b); Figure S7: Volcano plot comparing HCC tumors vs normal liver for PCM1 genes; Figure S8: Protein Network Analysis of PCM1; Figure S9: Protein–protein interaction network of PCM1; Table S1: Relationship with Scheuer score in GSE84044; Table S2: Overrepresentation among 176 gene panel via PANTHER (biological process); Table S3: Cibersort-based immune cell fractions; Table S4: GSEA results of the curated liver cancer related gene sets between PCM1-U and PCM1-D groups; Table S5: Genes that are significantly related to overall survival in GSE14520. Table S6: Common 500 gene sets enriched (GSEA) in GSE84044 and GSE83148; Table S7: PCM1 gene list (genes, probesets); Table S8: Differentially expressed genes between HCC tumors and normal liver; Table S9: Genes included in clusters 1–5; Table S10: Immunohistochemical evaluation of PCM1.
