# Supplementary material for: Transcriptomic Analysis of Hepatitis B Infected Liver for Prediction of Hepatocellular Carcinoma
Source: Biology (Basel). 2023 Jan 26;12(2):188. doi: 10.3390/biology12020188 (PMC9952979; doi:10.3390/biology12020188)
Supplement: Supplementary file 1 [file biology-12-00188-s001.zip › Sup. Figure S1.pdf]

Unsupervised clustering of CHB liver samples based on the most variant probesets

a

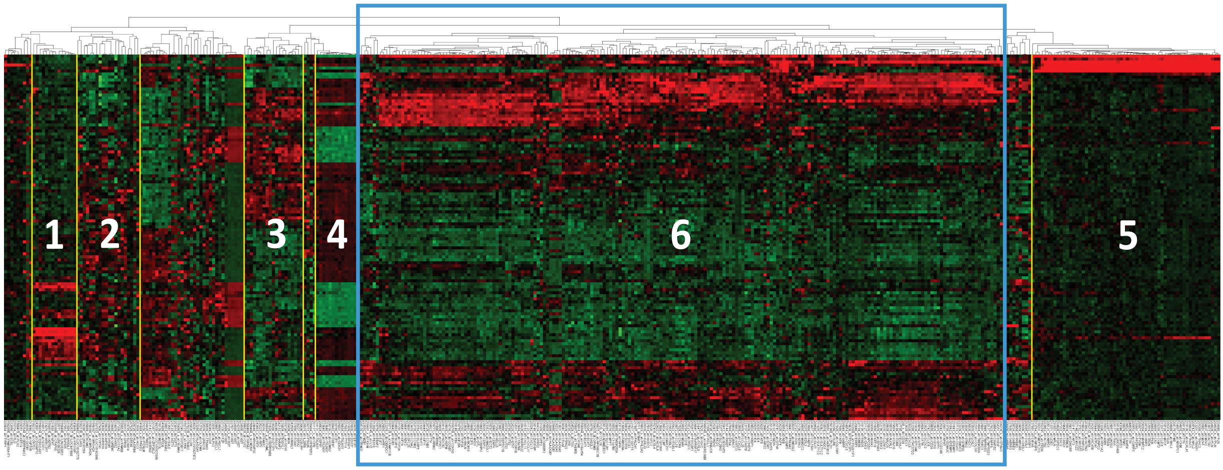

b

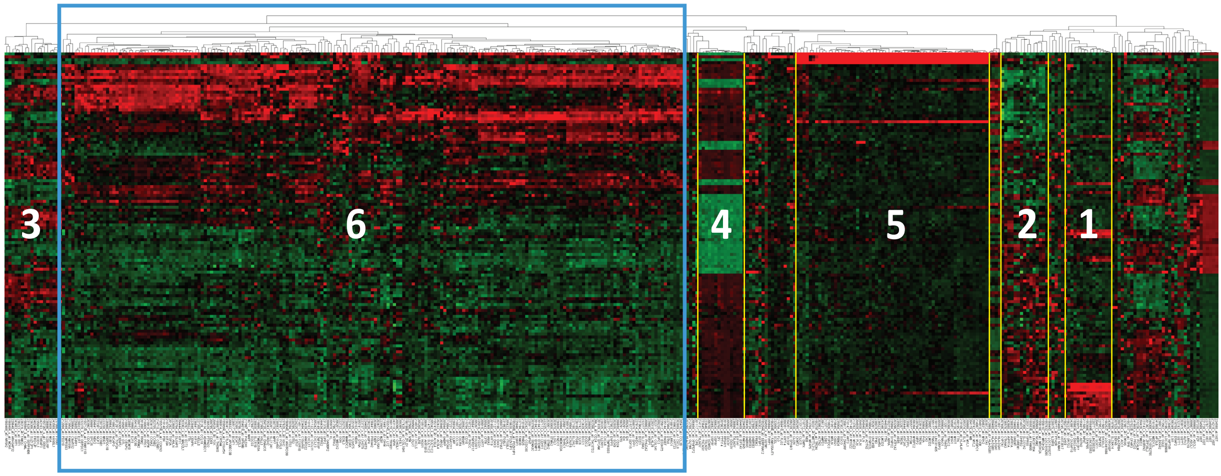

Supplementary Figure S1: Hierarchical clustering of CHB liver samples with the common most variant probesets in GSE83148 (a), GSE84044 (b). Expression of 476 probesets (columns) in 122 and 124 livers from CHB patients (rows) are shown. Red, black and green colors indicate high, moderate and low expression, respectively. Majority of the genes in the same numbered groups in two datasets overlapped. 6th group of genes shown in the blue box included 204 and 197 probesets that generated subgroups with high and low expression.
