# Supplementary material for: Transcriptomic Analysis of Hepatitis B Infected Liver for Prediction of Hepatocellular Carcinoma
Source: Biology (Basel). 2023 Jan 26;12(2):188. doi: 10.3390/biology12020188 (PMC9952979; doi:10.3390/biology12020188)
Supplement: Supplementary file 1 [file biology-12-00188-s001.zip › Sup. Figure S2.pdf]

Common probesets and genes identified in GSE83148 and GSE84044

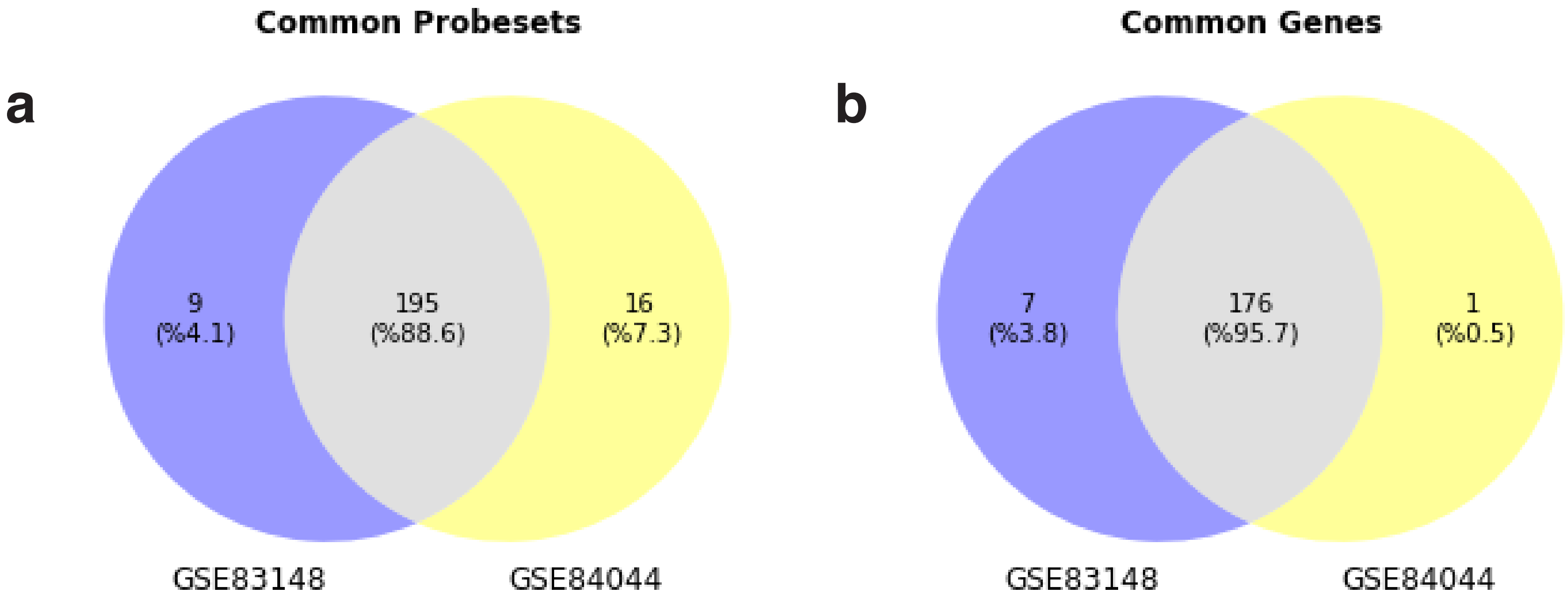

Supplementary Figure S2: Venn diagrams showing overlap of probesets (a) and genes (b) identified based on independent datasets (PCM1).
