# Supplementary material for: Transcriptomic Analysis of Hepatitis B Infected Liver for Prediction of Hepatocellular Carcinoma
Source: Biology (Basel). 2023 Jan 26;12(2):188. doi: 10.3390/biology12020188 (PMC9952979; doi:10.3390/biology12020188)
Supplement: Supplementary file 1 [file biology-12-00188-s001.zip › Sup. Figure S3.pdf]

Unsupervised clustering of independent datasets based on PCM1 signature

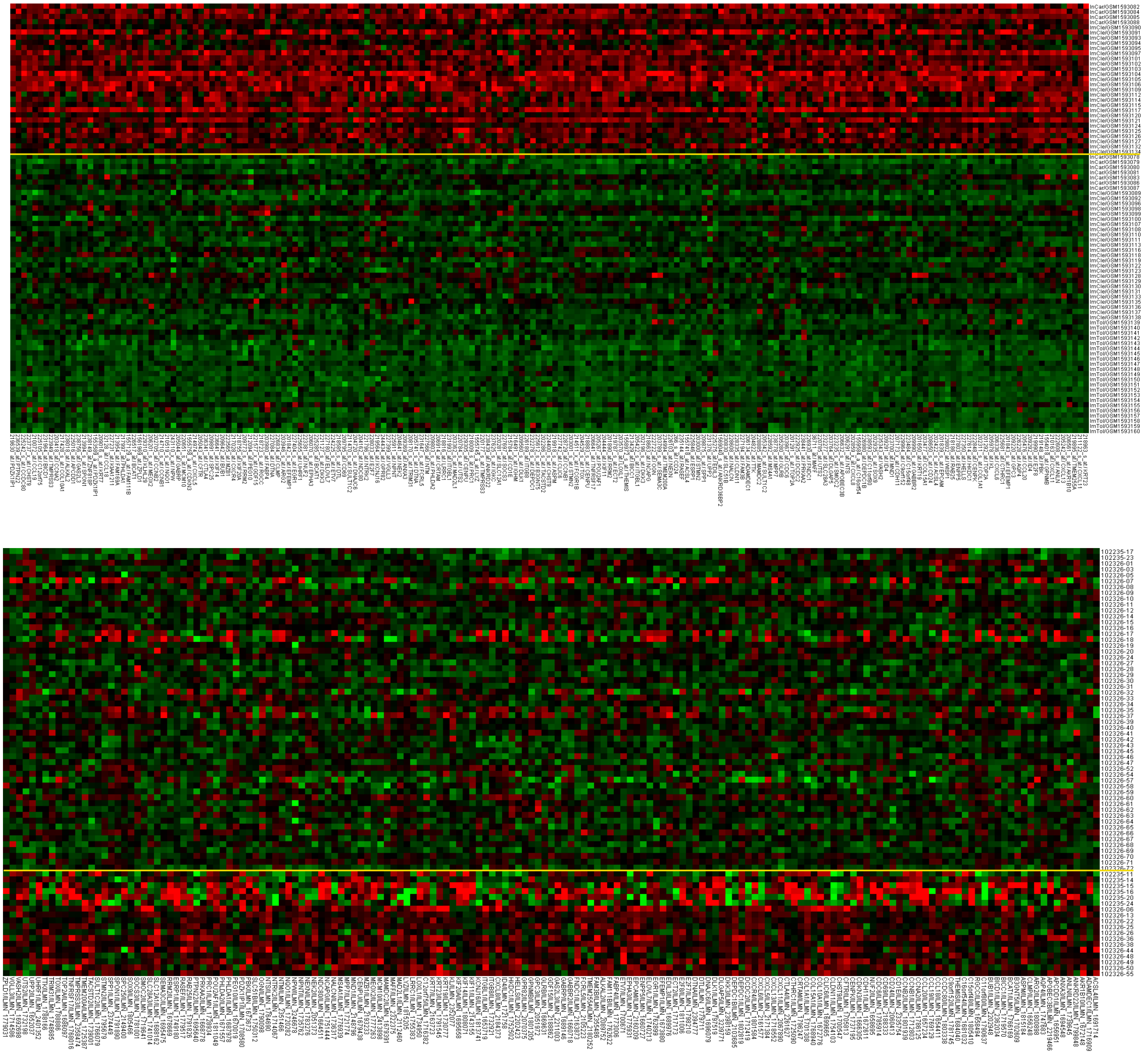

Supplementary figure S3: Hierarchical clustering of liver samples from CHB patients in GSE65359 (n=83) (a) and GSE83898 (n=73) (b) datasets. The complete list of PCM1 signature (195 probesets/176 genes) was used for the analysis of the GSE65359 dataset. 167 PCM1 genes available in the GSE83898 dataset were included in the analysis. For genes with multiple probesets, the probeset with the highest coefficient of variation was selected for GSE83898. Red, black and green colors indicate high, moderate and low expression, respectively. 29 and 54 patients were assigned to PCM1-U and PCM1-D groups in GSE65359, respectively. 18 and 55 patients were assigned to PCM1-U and PCM1-D groups in GSE83898, respectively. Groups are separated by the yellow line.
