# Supplementary material for: Transcriptomic Analysis of Hepatitis B Infected Liver for Prediction of Hepatocellular Carcinoma
Source: Biology (Basel). 2023 Jan 26;12(2):188. doi: 10.3390/biology12020188 (PMC9952979; doi:10.3390/biology12020188)
Supplement: Supplementary file 1 [file biology-12-00188-s001.zip › Sup. Figure S4.pdf]

Functional analysis of genes included in PCM1

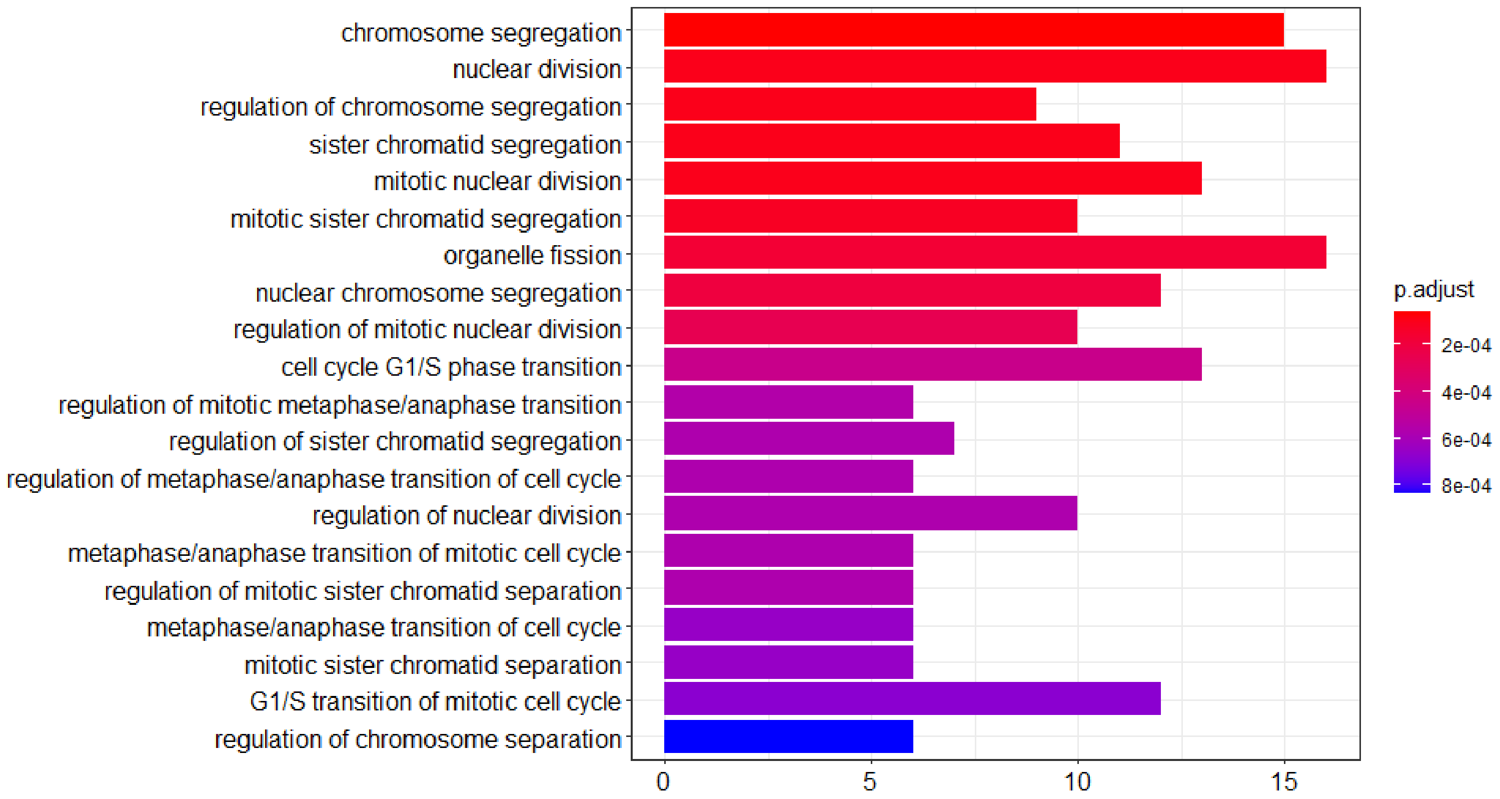

Supplementary Figure S4: Functional over-representation of 176 gene panel. Top 20 mechanisms with lowest enrichR based adjusted p value are shown.
