# Supplementary material for: Transcriptomic Analysis of Hepatitis B Infected Liver for Prediction of Hepatocellular Carcinoma
Source: Biology (Basel). 2023 Jan 26;12(2):188. doi: 10.3390/biology12020188 (PMC9952979; doi:10.3390/biology12020188)
Supplement: Supplementary file 1 [file biology-12-00188-s001.zip › Sup. Figure S5.pdf]

Expression of cell cycle related genes in PCM1-U and PCM1-D livers

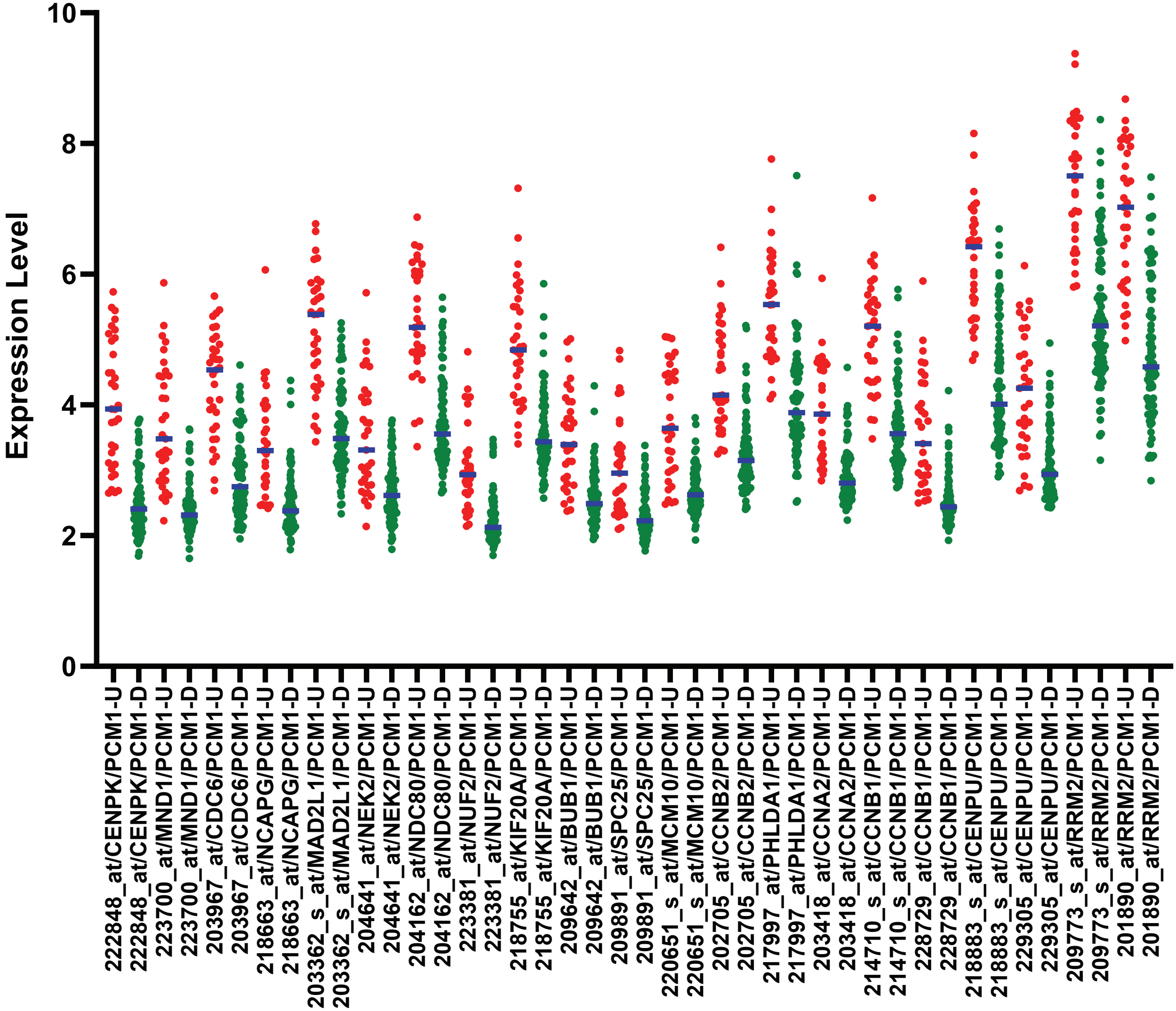

Supplementary Figure S5: Cell cycle related gene expression in PCM1-U (red) and PCM1-D (green) groups (GSE83148). Horizontal line indicates median.
