# Supplementary material for: Transcriptomic Analysis of Hepatitis B Infected Liver for Prediction of Hepatocellular Carcinoma
Source: Biology (Basel). 2023 Jan 26;12(2):188. doi: 10.3390/biology12020188 (PMC9952979; doi:10.3390/biology12020188)
Supplement: Supplementary file 1 [file biology-12-00188-s001.zip › Sup. Figure S6.pdf]

Enrichment of liver cancer related gene sets in PCM1-U

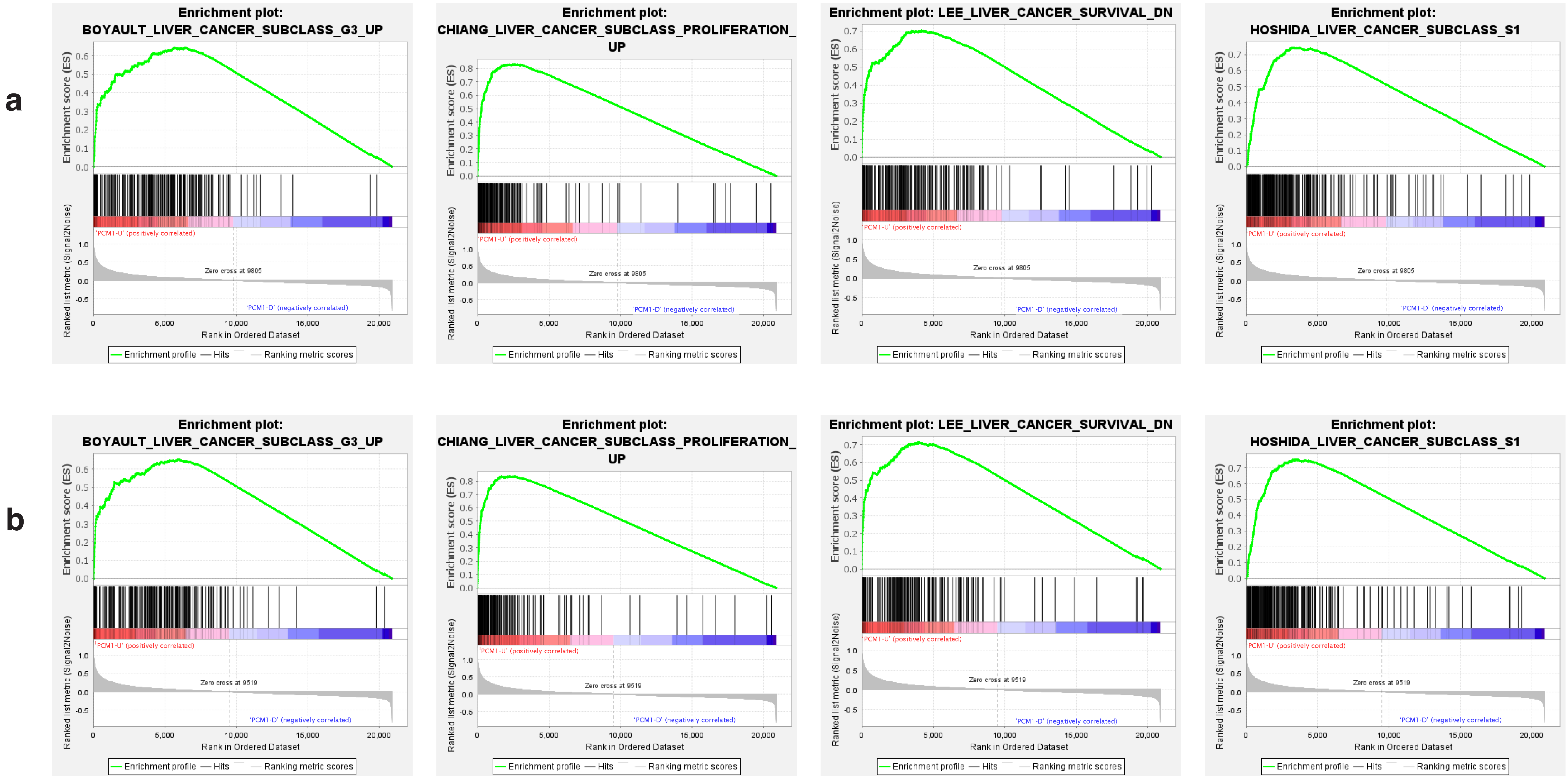

Supplementary figure S6: Enrichment plots of the curated liver cancer related gene sets in GSE83148 (a) and GSE84044 (b). PCM1-U and PCM1-D groups were compared.
