# Supplementary material for: Transcriptomic Analysis of Hepatitis B Infected Liver for Prediction of Hepatocellular Carcinoma
Source: Biology (Basel). 2023 Jan 26;12(2):188. doi: 10.3390/biology12020188 (PMC9952979; doi:10.3390/biology12020188)
Supplement: Supplementary file 1 [file biology-12-00188-s001.zip › Sup. Figure S7.pdf]

PCM1 gene expression in HCC tumors vs. normal liver

a

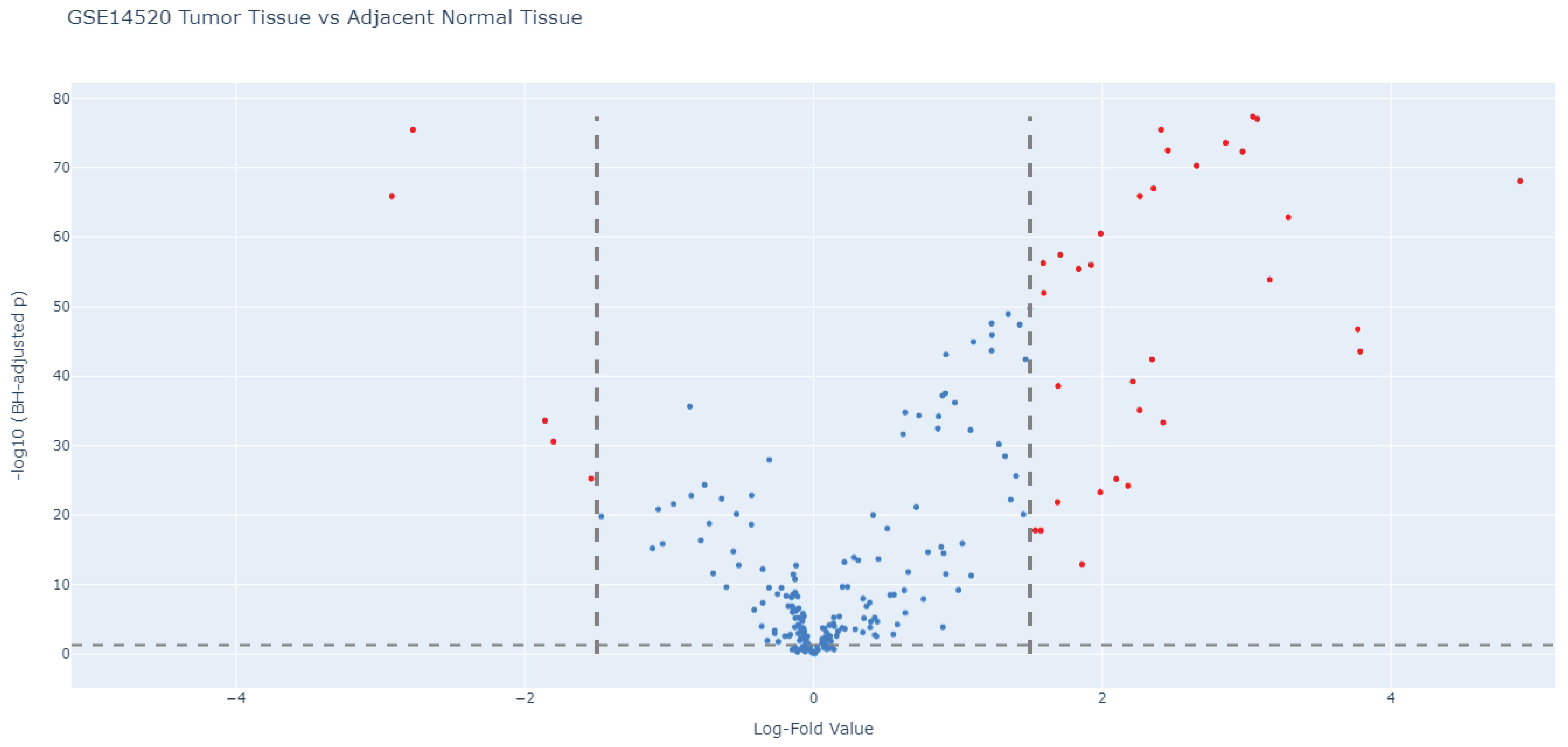

b

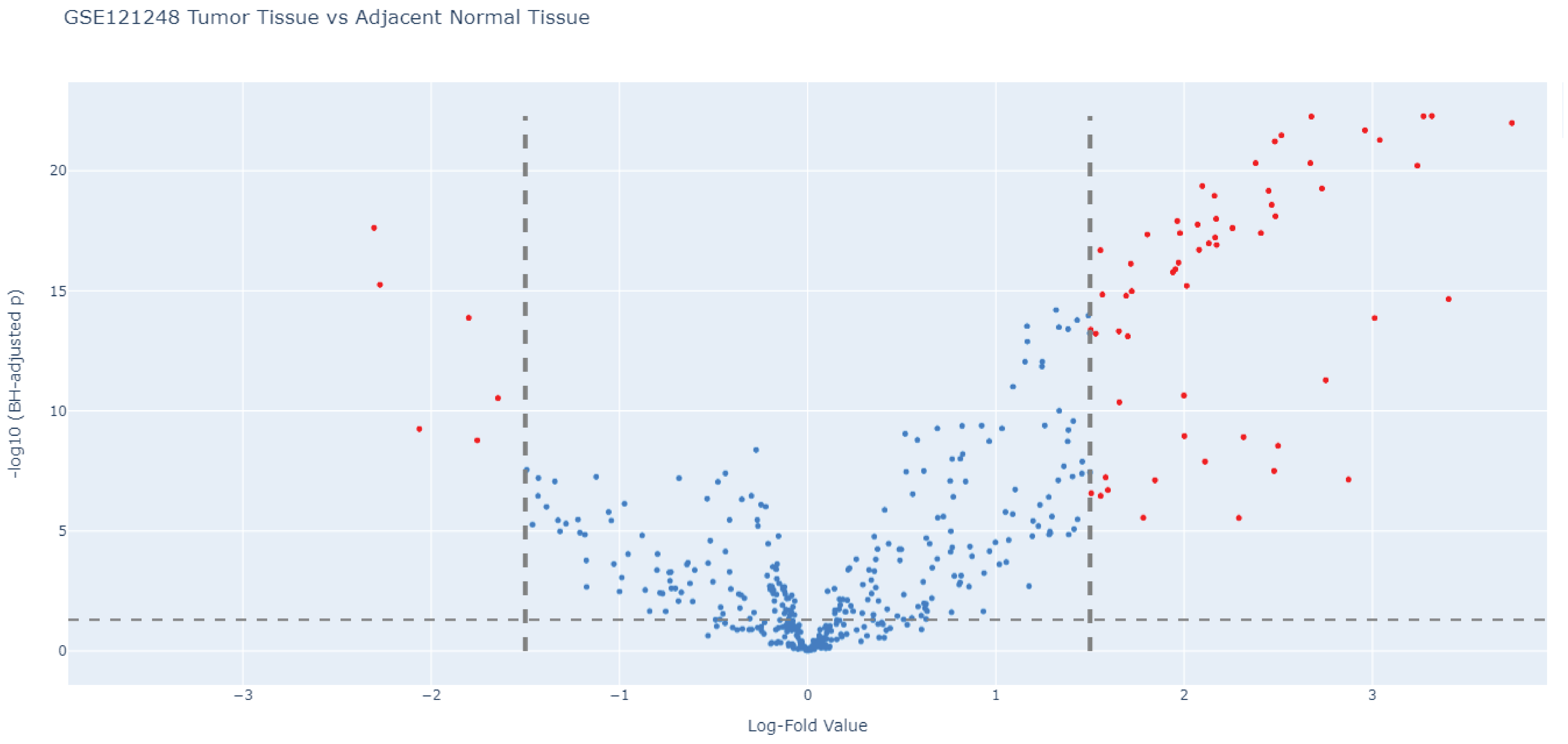

Supplementary Figure S7: Volcano plot comparing HCC tumors vs normal liver for PCM1 genes. Unpaired and paired t-test were per-formed in GSE121248 and GSE14520 for each probeset, respectively. Each dot represents one probeset. Log fold changes and Ben-jamini Hoechberg corrected p values are given in x and y axis, respectively.
