# Supplementary material for: Transcriptomic Analysis of Hepatitis B Infected Liver for Prediction of Hepatocellular Carcinoma
Source: Biology (Basel). 2023 Jan 26;12(2):188. doi: 10.3390/biology12020188 (PMC9952979; doi:10.3390/biology12020188)
Supplement: Supplementary file 1 [file biology-12-00188-s001.zip › Supp. Figure S8.pdf]

Protein Network Analysis of PCM1

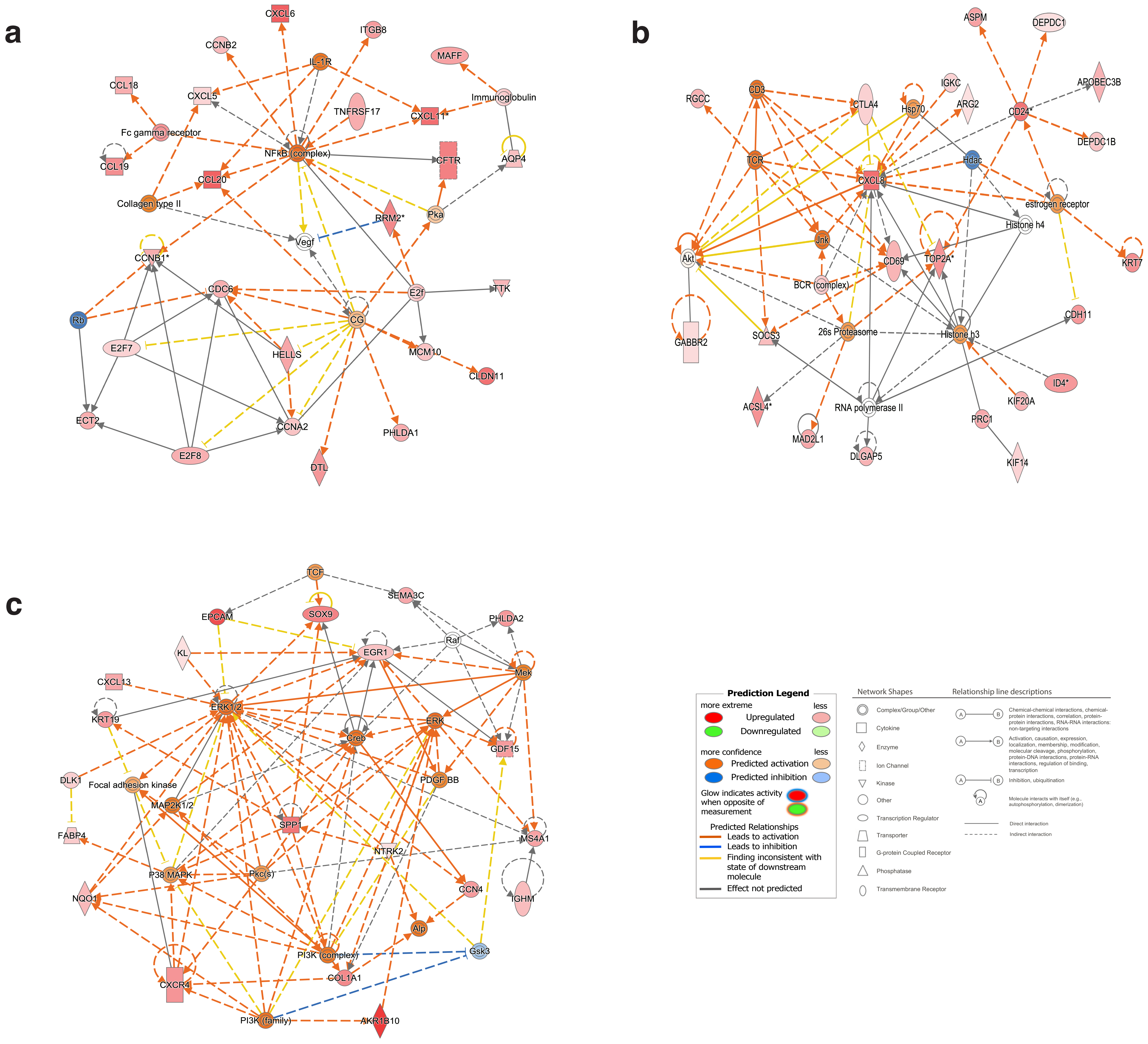

Supplementary Figure S8: Top molecular networks based on PCM1 generated through the use of IPA (QIAGEN Inc., https://digitalinsights.qiagen.com/IPA). Three networks with the highest number of focus molecules and the highest score (>30) are given. a, b and c show the top first, second, and the third network, respectively.
