# Supplementary material for: Transcriptomic Analysis of Hepatitis B Infected Liver for Prediction of Hepatocellular Carcinoma
Source: Biology (Basel). 2023 Jan 26;12(2):188. doi: 10.3390/biology12020188 (PMC9952979; doi:10.3390/biology12020188)
Supplement: Supplementary file 1 [file biology-12-00188-s001.zip › Supp. Figure S9.pdf]

Protein-protein interaction network of PCM1

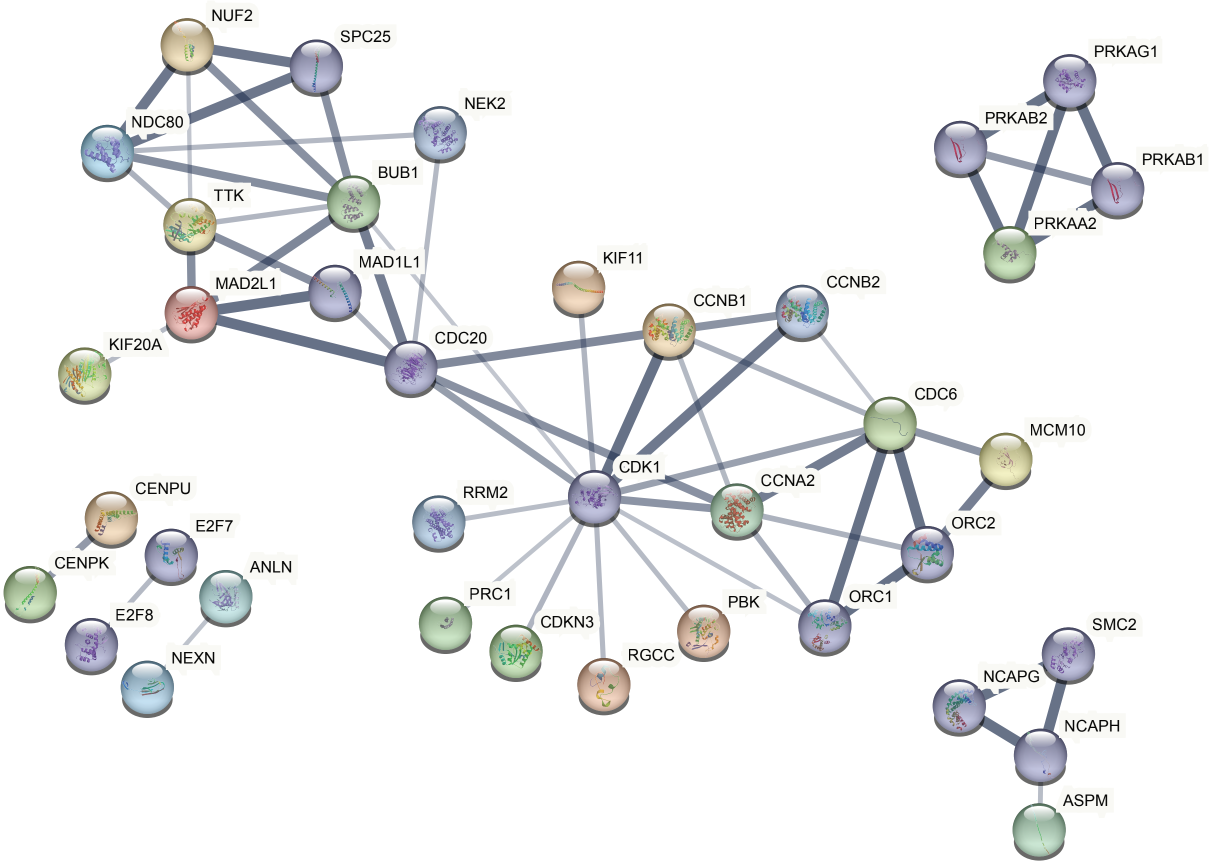

Supplementary figure S9: PPI network of proteins coded by PCM1 genes. 172 genes in PCM1 signature were used as input for network construction via STRING (https://string-db.org/). Line thickness indicates the strength of data support. The network was restricted to physical interactions with experimental evidence.
