# Supplementary material for: Transcriptomic Analysis of Hepatitis B Infected Liver for Prediction of Hepatocellular Carcinoma
Source: Biology (Basel). 2023 Jan 26;12(2):188. doi: 10.3390/biology12020188 (PMC9952979; doi:10.3390/biology12020188)
Supplement: Supplementary file 1 [file biology-12-00188-s001.zip › Supp. Tables S1-S5.pdf]

Supplementary Table S1: Relationship with Scheuer score in GSE84044

	Scheuer Score / Necroinflammatory Activity										
	**Grade 0**		**Grade 1**		**Grade 2**		**Grade 3**		**Grade 4**		p-value
	n	***%***	***n***	***%***	***n***	***%***	***n***	***%***	***n***	***%***	
**PCM1-D**	36	40.90%	28	31.80%	19	21.60%	3	3.40%	2	2.30%	<0.001
**PCM1-U**	1	2.80%	5	13.90%	15	41.70%	12	33.30%	3	8.30%	

Grade 0: portal/periportal activity (PPA) none or minimal, lobular activity (LA) none; Grade 1: PPA portal inflammation, LA inflammation but no necrosis; Grade 2: PPA mild piecemal necrosis, LA focal necrosis or acidophil bodies; Grade 3: PPA moderate piecemal necrosis, LA severe focal cell damage; Grade 4: PPA severe piecemal necrosis, LA damage includes bridging necrosis

	Scheuer Score / Fibrosis and Cirrhosis										
	**Grade 0**		**Grade 1**		**Grade 2**		**Grade 3**		**Grade 4**		p-value
	n	***%***	***n***	***%***	***n***	***%***	***n***	***%***	***n***	***%***	
**PCM1-D**	42	47.70%	18	20.50%	21	23.90%	5	5.70%	2	2.30%	<0.001
**PCM1-U**	1	2.80%	2	5.60%	12	33.30%	13	36.10%	8	22.20%	

Grade 0: Fibrosis (F) none; Grade 1: F enlarged, fibrotic portal tracts; Grade 2: F periportal or portal-portal septa but intact architecture; Grade 3: F fibrosis with architectural distortion but no obvious cirrhosis; Grade 4: F probable or definite cirrhosis

Supplementary Table S2 Overrepresentation among 176 gene panel via PANTHER (biological process)

GO Biological Process Complete	GO ID	Fold Enrichment	p value
regulation of mitotic metaphase/anaphase transition	GO:0030071	13.98	0.00822
chemokine-mediated signaling pathway	GO:0070098	13.2	0.00036
neutrophil chemotaxis	GO:0030593	12.52	0.00307
mitotic spindle organization	GO:0007052	12.04	0.00413
killing of cells of other organism	GO:0031640	11.3	0.00659
mitotic sister chromatid segregation	GO:0000070	10.93	0.000361
antimicrobial humoral immune response mediated by antimicrobial peptide	GO:0061844	10.12	0.00329
cell cycle G1/S phase transition	GO:0044843	8.41	0.015
mitotic prometaphase	GO:0000236	6.81	0.0253
mitotic cell cycle phase transition	GO:0044772	5.38	0.0111
cell division	GO:0051301	4.91	2.44E-05
regulation of cell adhesion	GO:0030155	3.47	0.00794
response to bacterium	GO:0009617	3.29	0.0324
regulation of cell population proliferation	GO:0042127	2.46	0.00547
regulation of multicellular organismal development	GO:2000026	2.19	0.0212
system development	GO:0048731	1.72	0.0152
positive regulation of cellular process	GO:0048522	1.6	0.0303

Supplementary Table S3: Cibersort based immune cell fractions

Cell Type	GSE83148			GSE84044			GSE65359			
	Fold Dif. (U-D)*	Adjusted p**	Rank	Fold Dif. (U-D)*	Adjusted p**	Rank	Fold Dif. (U-D)*	Adjusted p**	Rank	RANKSUM***
**Macrophages M1**	0.114921	4.97E-12	1	0.104081	1.20E-11	1	0.094086	3.12E-06	1.5	3.5
**T cells gamma delta**	0.074287	3.27E-05	3	0.072302	2.35E-06	2	0.083591	3.12E-06	1.5	6.5
**T cells regulatory (Tregs)**	-0.02179	2.83E-05	2	-0.0217	2.39E-05	4	-0.02074	0.000456	3	9
**Neutrophils**	-0.0148	0.000224	5.5	-0.01759	1.51E-05	3	-0.01499	0.00371	4	12.5
**Mast cells resting**	-0.02742	7.16E-05	4	-0.02007	0.000766	5	-0.01907	0.018482	5.5	14.5
**T cells CD4 naive**	-0.02232	0.000224	5.5	-0.0226	0.001022	6	-0.024	0.018482	5.5	17
**Mast cells activated**	0.016651	0.006123	7	0.015993	0.007288	10	0.021127	0.032138	7.5	24.5
**T cells CD8**	0.031053	0.016941	12	0.045602	0.003301	7	0.039411	0.039486	9	28
**Plasma cells**	0.017459	0.014917	11	0.019663	0.007288	10	0.023444	0.032138	7.5	28.5
**T cells follicular helper**	0.015767	0.008299	8	0.013245	0.011093	12	0.013039	0.134801	10	30
**NK cells activated**	-0.01729	0.010251	10	-0.01768	0.004743	8	-0.0133	0.184301	12	30
**Dendritic cells resting**	0.020762	0.008722	9	0.020295	0.007288	10	0.011482	0.232201	13.5	32.5
**Macrophages M0**	0.013035	0.073013	13	0.014576	0.026025	13	0.014452	0.159462	11	37
**Macrophages M2**	0.009749	0.184636	14	0.012304	0.086664	14	0.014553	0.232201	13.5	41.5
**B cells naive**	-0.00111	0.423248	15	-0.00101	0.429288	15	0.000743	0.915693	15	45

Cell types with 0 fractions in more than %50 of the samples' were excluded
*Fold difference was calculated by substraction of mean fraction of PCM1-D group from mean fraction of PCM1-U group
** Student's t-test based p values were adjusted using Benjamini Hochberg method.
*** "rank" indicates the rank of adjusted p value, "RANKSUM" is the sum of the ranks from three datasets

Supplementary Table S4: GSEA results of the curated liver cancer related gene sets between PCM1-U and PCM1-D groups

Enriched Geneset Name	GSE83148				GSE84044			
	ES	NES	NOM p-val	FDR q-val	ES	NES	NOM p-val	FDR q-val
**SHETH_LIVER_CANCER_VS_TXNIP_LOSS_PAM2**	0.661864	1.75598	0	0.005025	0.664639	1.774459	0	0
**SHETH_LIVER_CANCER_VS_TXNIP_LOSS_PAM1**	0.534732	1.743875	0	0.003141	0.545578	1.763564	0	0.00063
**BOYAULT_LIVER_CANCER_SUBCLASS_G3_UP**	0.637219	1.66648	0	0.009228	0.646725	1.690628	0	0.002579
**LEE_LIVER_CANCER_SURVIVAL_DN**	0.699302	1.657646	0.00202	0.007408	0.709841	1.67129	0	0.003189
**LEE_LIVER_CANCER_E2F1_UP**	0.71679	1.660932	0.002024	0.008136	0.738627	1.721376	0	0.001759
**HOSHIDA_LIVER_CANCER_SUBCLASS_S1**	0.741411	1.638775	0	0.006771	0.744788	1.664884	0	0.003453
**MARTORIATI_MDM4_TARGETS_FETAL_LIVER_UP**	0.42286	1.611421	0.004107	0.009433	0.428096	1.642822	0	0.004225
**HOSHIDA_LIVER_CANCER_SURVIVAL_UP**	0.776975	1.601924	0	0.009615	0.767106	1.622994	0	0.005642
**SHETH_LIVER_CANCER_VS_TXNIP_LOSS_PAM6**	0.575014	1.617768	0.005929	0.009366	0.583005	1.612019	0.003899	0.005062
**VILLANUEVA_LIVER_CANCER_KRT19_UP**	0.704284	1.571792	0.004016	0.013037	0.715163	1.60189	0	0.0059
**HOSHIDA_LIVER_CANCER_LATE_RECURRENCE_UP**	0.731084	1.585396	0.00198	0.010807	0.741896	1.632933	0	0.004946
**SERVITJA_LIVER_HNF1A_TARGETS_UP**	0.637742	1.556358	0.00409	0.013685	0.652335	1.579946	0.002008	0.006629
**CHIANG_LIVER_CANCER_SUBCLASS_CTNNB1_DN**	0.75385	1.548381	0.008048	0.015115	0.751845	1.571491	0	0.007393
**LEE_LIVER_CANCER_MYC_E2F1_UP**	0.742595	1.54601	0.004032	0.015113	0.761261	1.595766	0	0.006476
**LEE_LIVER_CANCER_DENA_UP**	0.748118	1.570467	0.004016	0.012566	0.746142	1.584371	0.002004	0.006736
**WOO_LIVER_CANCER_RECURRENCE_UP**	0.812587	1.527697	0	0.017641	0.810137	1.546168	0	0.010427
**CHIANG_LIVER_CANCER_SUBCLASS_PROLIFERATION_UP**	0.825271	1.48445	0.001996	0.025768	0.832444	1.479867	0	0.02598

Supplementary Table S5: Genes that are significantly related to overall survival in GSE14520

Gene	Probeset	HR	Cox p
**NTS**	206291_at	1.136602	0.000377
**SULT1C2**	211470_s_at	1.223927	0.00107
**MAFF**	36711_at	1.253452	0.001977
**PHLDA2**	209803_s_at	1.23706	0.002095
**SPP1**	209875_s_at	1.143059	0.003081
**SULT1C2**	205342_s_at	1.193248	0.004022
**BICC1**	213429_at	1.196197	0.007116
**KIF11**	204444_at	1.402865	0.007119
**CD24**	208650_s_at	1.165789	0.010313
**TOP2A**	201291_s_at	1.213884	0.010848
**GLRB**	205280_at	1.578242	0.011776
**CXCR4**	217028_at	1.256456	0.01545
**KRT19**	201650_at	1.176433	0.018503
**NCAPG**	218663_at	1.360636	0.018947
**KIF20A**	218755_at	1.286384	0.022774
**CD24**	266_s_at	1.154963	0.02361
**DLK1**	209560_s_at	1.067821	0.029951
**CXCL8**	202859_x_at	1.13132	0.034523
**ARG2**	203946_s_at	1.196763	0.035267
**DLGAP5**	203764_at	1.263921	0.042502
**CXCL13**	205242_at	0.798521	0.044972
**TOP2A**	201292_at	1.17908	0.048795
